# Expansion, persistence, and efficacy of donor memory-like NK cells infused for posttransplant relapse

**DOI:** 10.1172/JCI154334

**Published:** 2022-06-01

**Authors:** Roman M. Shapiro, Grace C. Birch, Guangan Hu, Juliana Vergara Cadavid, Sarah Nikiforow, Joanna Baginska, Alaa K. Ali, Mubin Tarannum, Michal Sheffer, Yasmin Z. Abdulhamid, Benedetta Rambaldi, Yohei Arihara, Carol Reynolds, Max S. Halpern, Scott J. Rodig, Nicole Cullen, Jacquelyn O. Wolff, Kathleen L. Pfaff, Andrew A. Lane, R. Coleman Lindsley, Corey S. Cutler, Joseph H. Antin, Vincent T. Ho, John Koreth, Mahasweta Gooptu, Haesook T. Kim, Karl-Johan Malmberg, Catherine J. Wu, Jianzhu Chen, Robert J. Soiffer, Jerome Ritz, Rizwan Romee

**Affiliations:** 1 Division of Transplantation and Cellular Therapies, Dana-Farber Cancer Institute, Harvard Medical School, Boston, Massachusetts, USA.; 2 Koch Institute for Integrative Cancer Research and Department of Biology, Massachusetts Institute of Technology, Cambridge, Massachusetts, USA.; 3 Center for Immuno-oncology, Dana-Farber Cancer Institute, Harvard Medical School, Boston, Massachusetts, USA.; 4 University of Milano-Bicocca, Monza, Italy.; 5 Department of Pathology and; 6 Department of Medical Oncology, Dana-Farber Cancer Institute, Harvard Medical School, Boston, Massachusetts, USA.; 7 Department of Data Science, Dana-Farber Cancer Institute/Harvard T.H. Chan School of Public Health, Boston, Massachusetts, USA.; 8 Department of Cancer Immunology, Institute for Cancer Research, Oslo University Hospital, Oslo, Norway.; 9 Institute for Clinical Medicine, The University of Oslo, Oslo, Norway.; 10 Center for Infectious Medicine, Department of Medicine, Karolinska Institutet, Solna, Sweden.

**Keywords:** Stem cells, Transplantation, Immunotherapy, NK cells, Stem cell transplantation

## Abstract

**Background:**

Responses to conventional donor lymphocyte infusion for postallogeneic hematopoietic cell transplantation (HCT) relapse are typically poor. Natural killer (NK) cell–based therapy is a promising modality to treat post-HCT relapse.

**Methods:**

We initiated this ongoing phase I trial of adoptively transferred cytokine-induced memory-like (CIML) NK cells in patients with myeloid malignancies who relapsed after haploidentical HCT. All patients received a donor-derived NK cell dose of 5 to 10 million cells/kg after lymphodepleting chemotherapy, followed by systemic IL-2 for 7 doses. High-resolution profiling with mass cytometry and single-cell RNA sequencing characterized the expanding and persistent NK cell subpopulations in a longitudinal manner after infusion.

**Results:**

In the first 6 enrolled patients on the trial, infusion of CIML NK cells led to a rapid 10- to 50-fold in vivo expansion that was sustained over months. The infusion was well tolerated, with fever and pancytopenia as the most common adverse events. Expansion of NK cells was distinct from IL-2 effects on endogenous post-HCT NK cells, and not dependent on CMV viremia. Immunophenotypic and transcriptional profiling revealed a dynamic evolution of the activated CIML NK cell phenotype, superimposed on the natural variation in donor NK cell repertoires.

**Conclusion:**

Given their rapid expansion and long-term persistence in an immune-compatible environment, CIML NK cells serve as a promising platform for the treatment of posttransplant relapse of myeloid disease. Further characterization of their unique in vivo biology and interaction with both T cells and tumor targets will lead to improvements in cell-based immunotherapies.

**Trial Registration:**

ClinicalTrials.gov NCT04024761.

**Funding:**

Dunkin’ Donuts, NIH/National Cancer Institute, and the Leukemia and Lymphoma Society.

## Graphical abstract

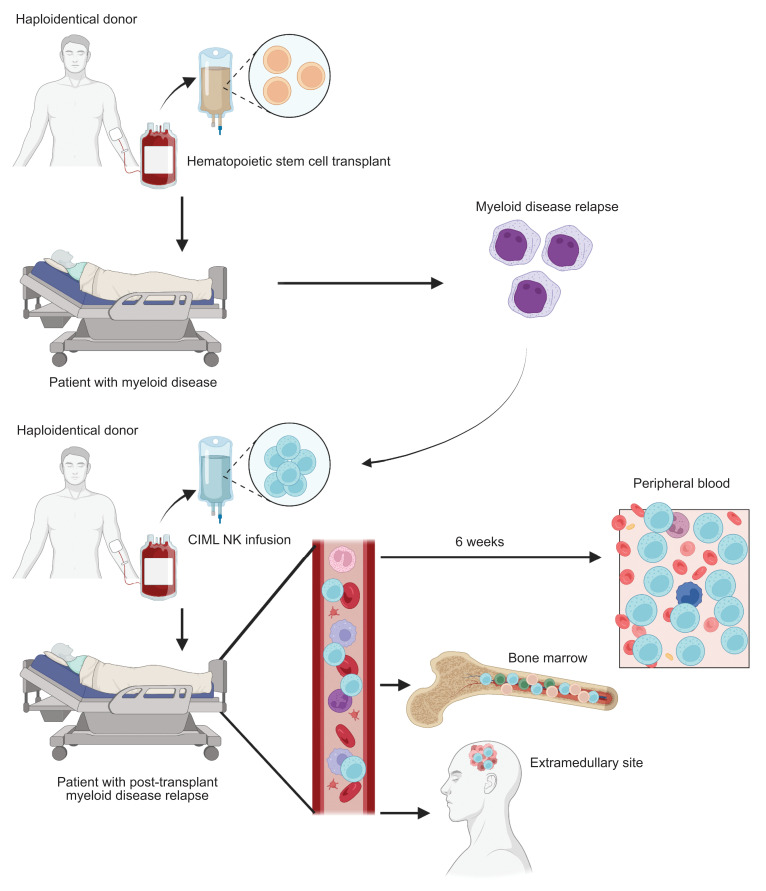

## Introduction

Relapse of acute myeloid leukemia (AML) occurs in up to 50% of patients after allogeneic hematopoietic stem cell transplant (HCT) and heralds an extremely poor prognosis (1). In several series of patients with AML who relapsed following HCT, the median overall survival was less than 6 months (2–3). Traditional approaches for the treatment of post-HCT relapse include the use of donor lymphocyte infusions (DLIs) or additional chemotherapy followed by a second HCT (1). While DLI is associated with remission rates of 10% to 25%, it is also associated with a high risk of moderate to severe graft-versus-host disease (GvHD), and long-term survival with this approach remains poor (4). With the recent increase in haploidentical HCT (haplo-HCT), whereby it is less clear whether the risk of GvHD due to DLI is outweighed by its efficacy (5–8), the treatment of posttransplant relapse must be improved.

The development of approaches for the management of post-HCT relapse of myeloid disease remains a high-priority area of research. Early post-HCT reconstitution of natural killer (NK) cells has been associated with a reduced risk of relapse in a number of retrospective studies, including in the T cell–depleted setting (9–11). NK cells are an attractive platform for the management of post-HCT relapse because they can mediate a graft-versus-leukemia effect without causing GvHD (11–15). The heterogeneity in clinical efficacy of predicted NK cell alloreactivity has been attributed in part to nonpersistence of NK cells following infusion, inhibition from Treg or myeloid suppressor cells, and tumor-induced NK cell anergy (16, 17). Strategies to overcome these barriers have focused on generating sufficient NK cell numbers that persist in vivo and retain their efficacy against potential target cells (18–20).

The incubation of donor-derived NK cells with interleukin-12 (IL-12), IL-15, and IL-18 generates a phenotype known as cytokine-induced memory-like (CIML) NK cells, which are capable of enhanced survival, expansion, and avoidance of anergy in vivo (21, 22). In preclinical studies, CIML NK cells exhibited a lower threshold of activation in response to cytokine restimulation as well as enhanced cytotoxicity against target leukemia cells (22). In a phase I trial of adoptively transferred CIML NK cells from a haploidentical donor in patients whose AML was relapsed/refractory and who never received a stem cell transplant, complete remission or complete remission with insufficient hematological recovery (CR/CRi) was attained in greater than 50% of subjects (21, 23). While exhibiting enhanced cytotoxicity against leukemia, the CIML NK cells were only detectable for 2 to 4 weeks in these treated patients due to HLA incompatibility. In contrast, adoptive transfer of CIML NK cells in the context of a syngeneic mouse model (24) resulted in persistence of functional cells for several months, arguing that their transfer into an immune-compatible environment can prolong their survival (25).

The prolonged persistence of functional CIML NK cells in an immune-compatible syngeneic mouse model supports their use in the post-HCT setting, a setting in which immune compatibility results from a common donor for both stem cells and subsequent adoptive transfer of NK cells (24). We therefore initiated a phase I trial of CIML NK cells to treat patients with relapsed myeloid malignancies after haplo-HCT. Here we describe the detailed characterization of the CIML NK cells in vivo in the first 6 patients treated on this trial, including transcriptome and proteome analysis at single-cell resolution.

## Results

### Adoptive transfer of donor-derived CIML NK cells is safe and associated with clinical responses.

**Figure 1:**
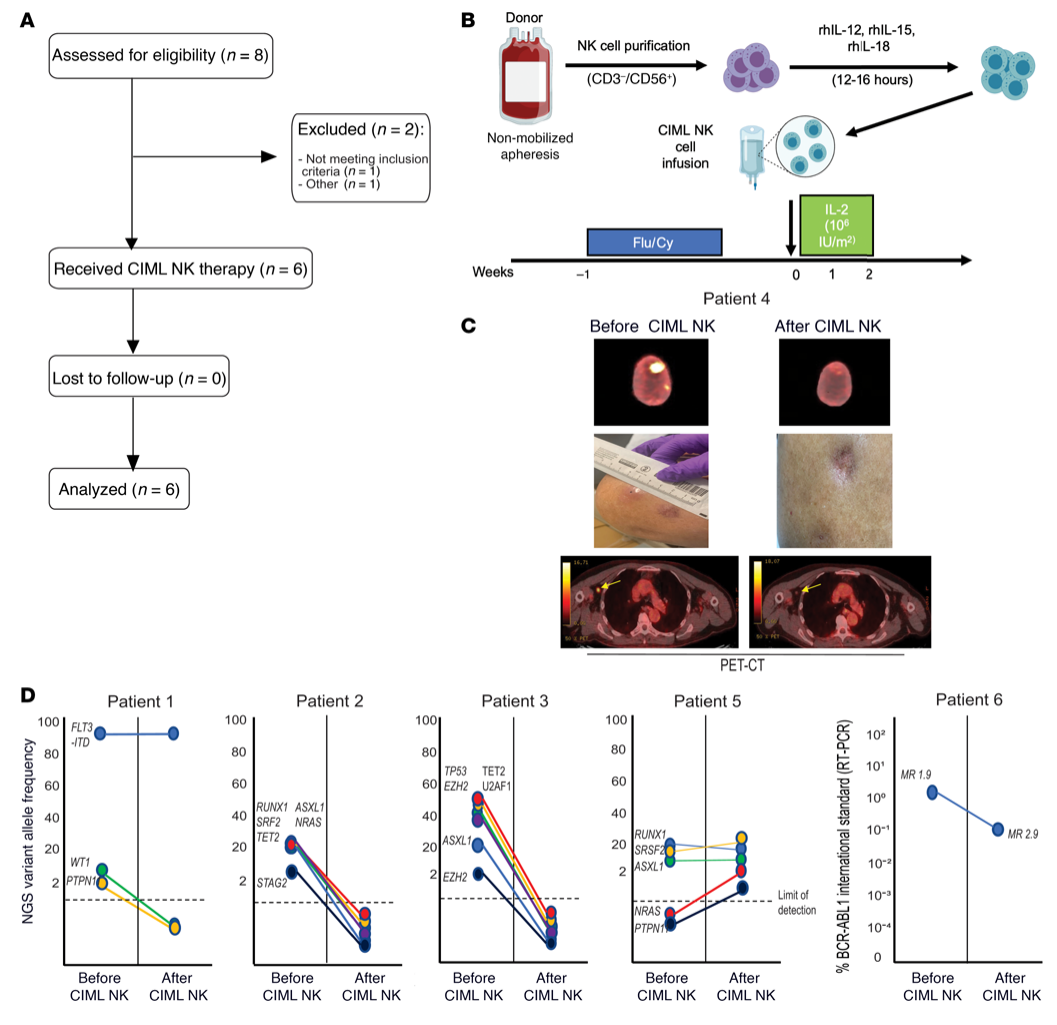
Phase I trial schema and clinical responses. (**A**) CONSORT flow diagram for the patients evaluated for the phase I trial. (**B**) Schema describing the generation of CIML NK infusion product and trial therapy. For each patient, the same donor who had previously given stem cells underwent nonmobilized apheresis of peripheral blood followed by CD3 depletion and CD56-positive selection. The resulting product was incubated with a cytokine cocktail for 12–16 hours, washed, and infused into the lymphodepleted patient. Following NK cell infusion, low-dose IL-2 was administered subcutaneously every other day for 7 doses. (**C**) Disease response of the BPDCN patient who had entirely extramedullary disease. Shown is the PET-CT imaging of the 2 identified lesions in the scalp with the corresponding visual evaluation of these lesions, and the active axillary lymph node. (**D**) Next-generation sequencing (NGS) or RT-PCR was used to track individual mutations or transcripts before and after treatment with the CIML NK cells. Patients 2 and 3 exhibited marrow CR (for MDS) and CR without minimal residual disease (for AML) on the day +28 assessment, respectively, while patient 1 exhibited a morphologic leukemia-free state with persistently detectable *FLT3*-ITD. Patient 5 had progressive disease as per ELN 2017 criteria for AML. Patient 6 exhibited a reduction in BCR-ABL1 transcripts after CIML NK infusion as measured with quantitative RT-PCR, but had persistent disease suggesting refractoriness to therapy.

Six patients whose myeloid disease relapsed after haplo-HCT were treated with a donor-derived CIML NK cell product as part of a phase I clinical trial (Figure 1, A and B). These included 3 with AML, 1 with myelodysplastic syndrome (MDS), 1 with blastic plasmacytoid dendritic cell neoplasm (BPDCN), and 1 with CML in blast crisis (patient characteristics are reported in Supplemental Table 1; supplemental material available online with this article; https://doi.org/10.1172/JCI154334DS1). As per trial inclusion criteria, none of the patients received any prior DLI, nor any other therapy concurrent with the CIML NK cell infusion. Patients tolerated the CIML NK cell infusion and expansion well, with fever as the most common side effect (temperature ranging from 38.1°C to 39.3°C) during the 12 days of IL-2 administration. One patient developed grade 2 cytokine release syndrome (CRS) 7 days after CIML NK cell infusion and was treated with tocilizumab. Four of 6 patients developed pancytopenia after CIML NK infusion. In 2 cases the pancytopenia was prolonged and these patients received CD34^+^ cell–selected stem cell boosts from their original donors 5 and 6 weeks after CIML NK therapy, respectively. Both patients responded to the stem cell boosts, with neutrophil recovery evident within 9 to 14 days after boost (Supplemental Figure 1). No patient developed any evidence of acute or chronic GvHD. One patient (patient 6) experienced a transient increase in alanine aminotransferase and aspartate aminotransferase while on IL-2, resulting in premature discontinuation of IL-2 (the patient received a total of 2 doses).

All patients were evaluable at the day +28 response assessments following infusion of CIML NK cells, and 4 of 6 patients attained a response to therapy. Three of 6 patients met European LeukemiaNet (ELN) 2017 criteria for CR in AML or International Working Group criteria for marrow CR in MDS (26, 27). These included patient 4 (BPDCN patient with entirely extramedullary disease relapse) who achieved a PET-negative CR (confirmed by tumor site biopsy) (Figure 1C), while patient 2 (with MDS and multiple pathogenic mutations) and patient 3 (with AML and pathogenic *TP53* mutation) had no detectable residual disease by next-generation sequencing after CIML NK cell therapy (Figure 1D). Patient 1, with FMS-like tyrosine kinase 3 internal tandem duplication–positive *FLT3*-ITD^+^) AML, attained a morphologic leukemia-free state with clearance of some of the pathogenic mutations present at the time of post–haplo-HCT relapse but had a persistent *FLT3*-ITD mutation on day +28 bone marrow assessment. Patients 5 and 6 exhibited disease refractoriness on the day +28 response assessment.

### CIML NK cell infusion is associated with rapid and prolonged expansion in the peripheral blood NK cell compartment without changes in Treg numbers.

**Figure 2:**
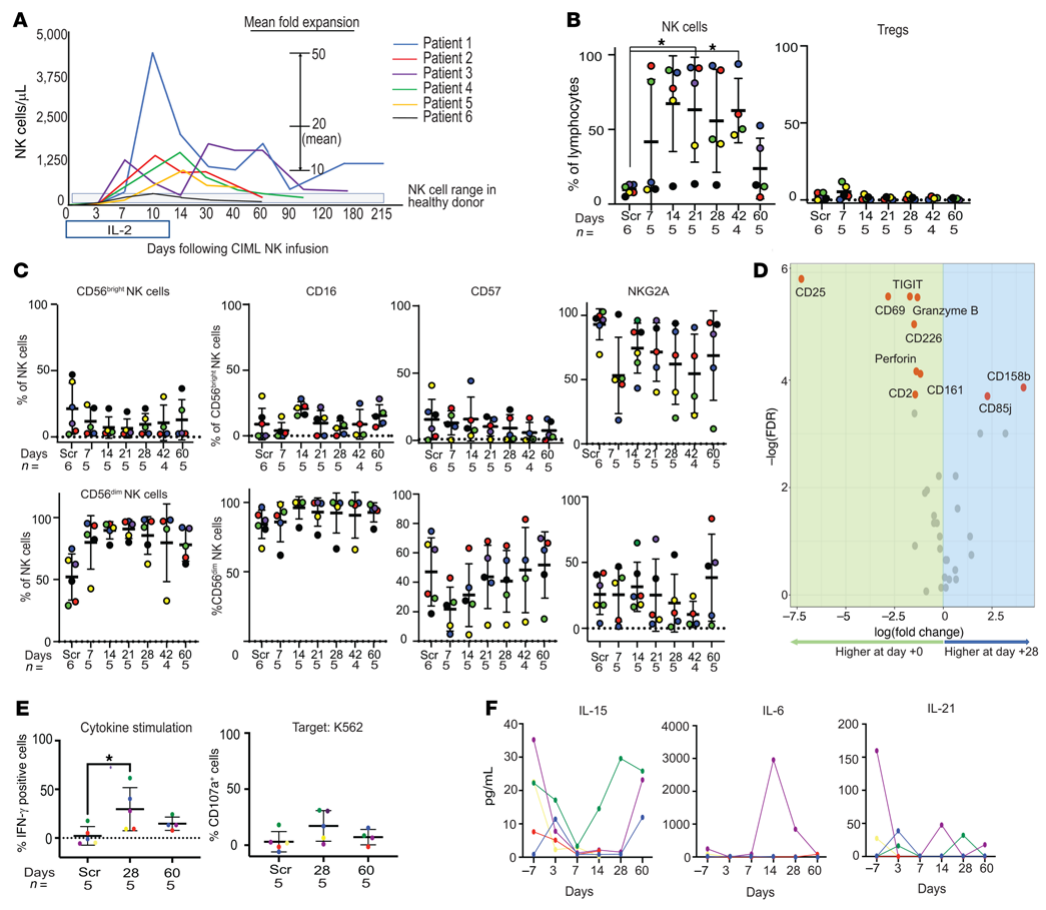
Rapid expansion of NK cells follows infusion of CIML NK product into the patients. (**A**) Mean NK cell counts/μL of peripheral blood determined from the lymphocyte count. (**B**) Flow cytometry was used to evaluate NK cells in the peripheral blood as a proportion of total lymphocytes. Data presented are CD56^+^CD3^–^ cells as a percentage of total lymphocytes, mean ± SD. Right: Tregs (CD3^+^CD4^+^CD25^+^CD127^–^) cells as a percentage of total lymphocytes, mean ± SD. The value for each patient is shown with a colored dot, and the coloring scheme corresponds to that in panel **A**. *P < 0.05 by Mann-Whitney *U* test, with significance adjusted by Holm’s method for multiple comparisons. (**C**) Flow cytometry–based evaluation of key markers distinguishing the CD56^dim^ NK cell population from the CD56^bright^ NK cell population. There was no significant difference in expression of markers between time points as determined by the Mann-Whitney *U* test. (**D**) Differential expression analysis of mass cytometry markers in the predominant CD56^dim^ clusters between day 0 (infusion product) and day +28 after infusion. Markers labeled in red are differentially expressed (Wilcoxon’s signed-rank test, *P <* 0.05). (**E**) Functional characterization of the expanded NK cell compartment using both cytokine stimulation and coculture with K562 target cells at an E:T ratio of 5:1. The *y* axis shows percentage expression of the indicated marker relative to the corresponding unstimulated control, and the dashed line represents the same functional assays applied to healthy donor control PBMCs. In **B**, **C**, and **E**, the screening time point (Scr) refers to endogenous patient NK cells prior to infusion. *P < 0.05 by Mann-Whitney *U* test. (**F**) Measurement of plasma cytokines following CIML NK cell infusion. The *x* axis of each plot shows days relative to CIML NK cell infusion (day 0).

Following CIML NK cell infusion, NK cell numbers dramatically expanded to a median peak of 10-fold (range 10- to 50-fold; Figure 2A). NK cells constituted the dominant lymphocyte subset, with increased NK cell numbers persisting for up to several months beyond the last dose of IL-2 (day +12) in these patients (Figure 2B). T cells constituted a minority of peripheral blood mononuclear cells (PBMCs) in the peripheral blood compartment after NK cell expansion. There was no significant increase in the Treg numbers (Figure 2B); CD8^+^ T cells were the major subset of T cells present in the first weeks after CIML NK infusion (Supplemental Figure 2).

During their expansion, NK cells were predominantly CD56^dim^ cells (Supplemental Figure 3) and displayed classical NK cell maturity markers, including KIR and CD57 (Figure 2C and Supplemental Figure 3). Expansion of the NK cell population on day +28 following infusion of CIML NK cells was associated with reduced expression of several markers, including CD2, TIGIT, CD161, CD226, granzyme B, and perforin when compared with NK cells in the infusion product (Wilcoxon’s rank-sum test *P <* 0.05; Figure 2D).

Functional evaluation of infused NK cells on day +28 and day +60 after infusion was performed by cytokine (IL-12 plus IL-18) stimulation or coculture with K562 cells and comparing with NK cells from the screening time point before any therapy. On day +28 following infusion, IFN-γ expression was increased in response to cytokine stimulation (Figure 2E). CD107a expression was upregulated after coculture with K562 on day +28 compared with the time of screening in patients 1, 2, and 4, but not in other patients.

Evaluation of the expression of a panel of endogenous cytokines potentially associated with NK cell proliferation revealed the lack of increase in IL-15 or IL-21 at the time of exogenous IL-2 administration, although IL-15 appeared to increase in patient 4 after day +7 (Figure 2F). Patient 3, who developed CRS after NK cell infusion, had a corresponding increase in IL-6 and TNF-α, supporting the diagnosis (Supplemental Figure 4).

### Longitudinal evaluation of the NK cell compartment after CIML NK cell infusion reveals expansion and persistence of both adaptive and nonadaptive NK cell subpopulations.

To be able to longitudinally evaluate the NK cell compartment in all 6 patients after CIML NK infusion, we defined the NK cell populations present in the infusion products using flow cytometry, a 39-marker mass cytometry panel, and single-cell RNA sequencing (scRNA-seq). All infusion products met product release criteria (≥70% CD3^–^CD56^+^ and <3 × 10^5^/kg CD3^+^ cells) and had a high purity of NK cells. On mass cytometric analyses, the NK cell subpopulations that could be identified included adaptive CD56^dim^ NK cells, those that expressed NKG2A, both CD16^+^ and CD16^–^ fractions, and those that expressed CD8 (Supplemental Figure 5, A and B). To deconvolute each patient’s contribution to these clusters (patient 6 was excluded from this specific analysis as they received only 2 out of planned 7 IL-2 doses), we used the following set of markers: CD56, CD3, NKG2A, IL-7Ra, KIR2DL1, NKG2C, NKp46, NKp30, and Ki67. The resulting clusters delineated several identifiable NK cell populations in the infusion product, including adaptive NK cells (NKG2C^+^KIR^+^), CD56^bright^ NK cells (CD56^hi^NKG2A^hi^KIR2DL1^–^), and CD56^dim^NKG2A^lo^ NK cells (Supplemental Figure 5C). The infusion products in patients 1 and 2 had a large subpopulation of adaptive NK cells expressing both NKG2C and KIR. Patients 3 and 4, on the other hand, had a subpopulation with a higher level of CD56 and NKG2A expression and a relatively low KIR and NKG2C expression, consistent with CD56^bright^ NK cells that were largely absent in the other patients (Supplemental Figure 5C). Additional characterization of the CIML NK infusion products with scRNA-seq also identified several CD56^dim^ and adaptive NK cell populations, as well as additional CD56^bright^ NK cell subpopulations that were not detected by mass cytometry (Supplemental Figure 6A). The CD56^dim^ subpopulations in the infusion product had a similar level of expression of NK cell lineage markers, with the only noticeable differences being in the expression of *FCER1G* and *NCR3* genes (Supplemental Figure 6B).

To investigate the specific phenotypic imprint of the CIML NK cell preparation, we compared the CIML NK cell infusion product with the original resting donor-derived repertoire from screening samples using mass cytometry. The latter samples corresponded to the allogeneic donor-derived NK cell compartment within the recipient following HCT, but prior to receiving the infusion product. Compared with the resting donor NK cell repertoire, CIML NK cells exhibited a cytokine-stimulated signature, including a higher expression of CD25 and CD69, and a trend toward higher expression of CD161 that is thought to mark cytokine-responsive NK cells (Supplemental Figure 7 and ref. 28). Expression of NKG2D and TRAIL was lower on the infusion products from all patients compared with the screening time point. The immunomodulatory markers TIM3 and TIGIT were more highly expressed on the infusion product than at screening (Supplemental Figure 7).

**Figure 3:**
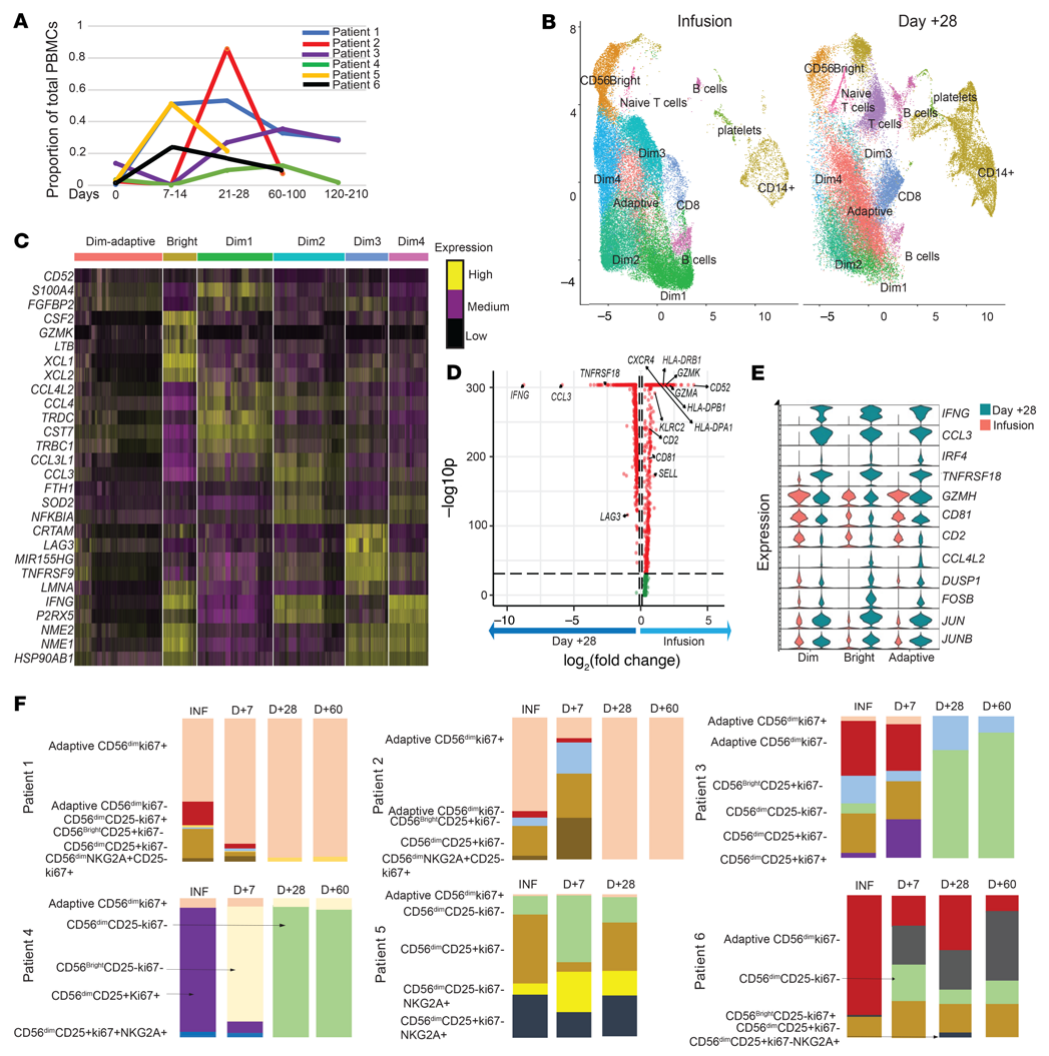
Evaluation of subpopulations of NK cells present in the infusion product over time. (**A**) Median proportion of total PBMCs that were CD56^+^CD3^–^ at the indicated time points. Days after infusion of the CIML NK product are indicated above the time point labels. (**B**) UMAP of NK cell clusters defined with single-cell RNA sequencing at day +28 compared to the infusion product for all patients whose CIML NK cells expanded. The CD56^dim^ NK cell subpopulations that persist include Dim1, Dim2, CD56^bright^, and adaptive NK cells. (**C**) Identification of defined NK cell subpopulations based on transcriptional profiling reveals the expansion of several CD56^dim^ subpopulations and adaptive CD56^dim^ NK cell populations. The heatmap shows the top 5 differentially expressed genes in each NK cell cluster when compared with other clusters using Wilcoxon’s rank-sum test (log[fold change] threshold = 0.25). (**D**) Volcano plot showing the top differentially expressed genes in all CD56^dim^ clusters between infusion and day +28; fold change cutoff = 0.5, *P*-value cutoff = 10 × 10^–32^. Differential gene expression was determined using the nonparametric Wilcoxon’s rank-sum test. (**E**) Violin plot to show the expression of select genes within the CD56^dim^, CD56^bright^, and adaptive CD56^dim^ NK populations at infusion (red) and day +28 (green) time points. Shown are the most differentially expressed markers as well as genes associated with NK cell activation. (**F**) Evaluation of NK cell subpopulations over time as a proportion of total NK cells in each of the clinical trial patients treated with CIML NK cell therapy. Key subpopulations include adaptive NK cells (CD56^+^CD3^–^CD57^+^KIR^+^NKG2C^+^FcεRI^–^), CD56^bright^ NK cells (CD56^hi^CD3^–^NKG2A^+^IL-7R^+^), CD56^dim^NKG2A^+^ NK cells, and CD56^dim^Ki67^+^ NK cells, among others. The sample time points for each patient are labeled. INF, at time of infusion.

Phenotypic evaluation over time of the NK cell subpopulations present in the infusion product showed their persistence on day +28 after CIML NK infusion (Figure 3). Patients 1, 3, and 4 exhibited the longest persistence of NK cell subpopulations phenotypically matching those that were infused (Figure 3A). We used scRNA-seq to compare major clusters present in the infusion product and at the day +28 time point. Distinct clusters of CD56^dim^, adaptive CD56^dim^, and CD56^bright^ NK cells were identified, alongside several nonadaptive CD56^dim^ NK cell clusters (Figure 3B). Characterization of the nonadaptive CD56^dim^ clusters revealed distinct expression of the chemokine genes *CCL4* (Dim1) and *CCL3* (Dim2) in 2 of the clusters present on day +28. *IFNG* was shown to be more highly expressed in Dim2 and Dim4, while the cells in Dim3 expressed a higher level of *LAG3* (Figure 3C).

We then compared the gene expression in CD56^dim^ clusters at infusion with those present on day +28 after CIML NK cell infusion. These genes included *KLRC2*, *CD52*, and *IFNG*, the expression of which is typically associated with adaptive NK cells (Figure 3D). Expression of the *IFNG* gene was increased in all NK cell subpopulations on day +28 when compared with the infusion time point (Figure 3E). Additional genes associated with NK cell activation, including *JUN*, *FOSB*, and *DUSP1*, as well as the chemokine *CCL3* had increased expression in each of the major NK cell subpopulations (Figure 3E). The top 50 differentially expressed genes in the CD56^dim^ and CD56^bright^ NK clusters at infusion compared with day +28 are summarized in Supplemental Tables 2 and 3.

We then longitudinally evaluated the transcriptionally defined NK cell populations using mass cytometry in all the trial patients on days +7, +28, and +60 (Figure 3F). The persistence of NK cell clusters identified in the infusion product was most notable in patients 1, 2, and 5. Patients 3 and 4 both exhibited a dominance of NK clusters on day +28 and day +60 that constituted a minor portion of the infusion product. Patient 6 had a poor expansion of infused CIML NK cells. The majority of CD25^+^ NK cell subsets were absent after day +7 in all patients. Furthermore, the majority of infused CIML NK cells that expanded by day +28 and persisted to day +60 were Ki67^–^.

### CMV reactivation drives the expansion of adaptive NK cells, but not other NK cell subsets.

**Figure 4:**
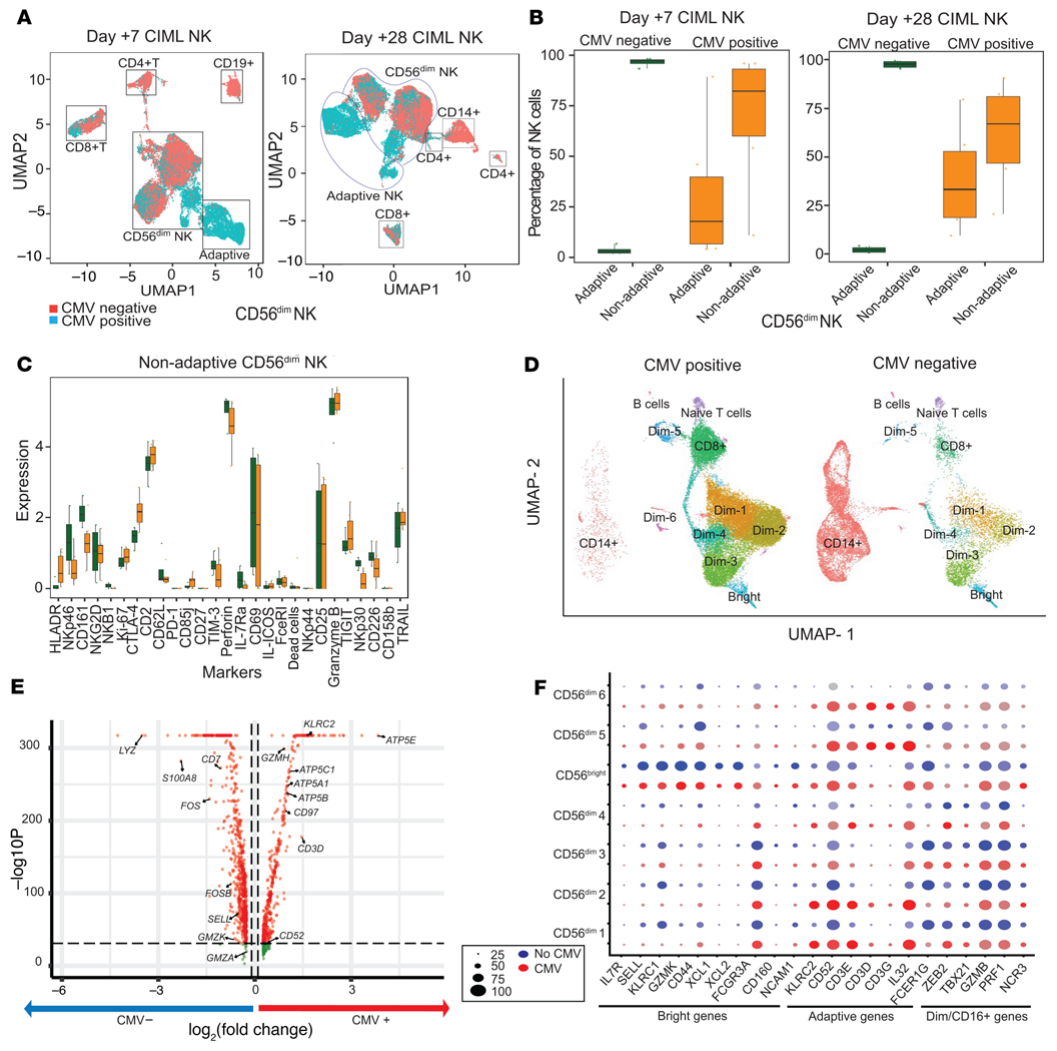
Distribution of adaptive and nonadaptive CD56^dim^ NK cell populations and markers in the CMV+ (*n* = 3) and CMV– (*n* = 2) groups. (**A**) UMAP of PBMC subpopulations identified by mass cytometric analysis showing both CMV+ (light green) and CMV– (red) cells on both day +7 and day +28. (**B**) Distribution of adaptive and nonadaptive CD56^dim^ NK cells in the CMV– (green) and CMV+ group (orange) on day +7 and day +28 after CIML NK cell infusion. (**C**) Differential expression of markers on the nonadaptive CD56^dim^ NK cell subpopulations on day +28 after CIML NK cell infusion shows no differences in this subpopulation between CMV+ and CMV– groups. *P < 0.05 by Wilcoxon’s rank-sum test, with significance adjusted for multiple comparisons (43). (**D**) UMAP of PBMC subpopulations in CMV+ and CMV– patients evaluated with scRNA-seq on day +28 after CIML NK cell infusion. The adaptive Dim1, Dim2, Dim5, and Dim6 subpopulations are expanded predominantly in the CMV+ group, while the Dim3 and Dim4 subpopulations are comparable between groups. (**E**) Volcano plot to show the most differentially expressed genes in the adaptive CD56^dim^ NK cell clusters between CMV+ and CMV– groups (*P*-value cutoff = 10 × 10^–32^, fold change cutoff = 0.1). The MAST test was used to determine differentially expressed markers between the 2 groups (log[fold change] threshold = 0.25). (**F**) Dot plot showing genes defining the individual clusters identified in the CMV+ and CMV– patient groups on day +28 after infusion. Subsets of genes corresponding to CD56^bright^, adaptive CD56^dim^, and nonadaptive CD56^dim^CD16^+^ NK cells are indicated.

Three of the patients had a low-titer CMV viremia during this trial, although none of these patients developed CMV disease and/or needed treatment. To address the potential impact of CMV reactivation on the expanding NK cell compartment, we correlated absolute NK cell numbers with CMV reactivation (Supplemental Figure 8, A and B) and compared the expansion of NK cells between patients 1, 2, and 3 who had CMV reactivation (CMV+ group), and patients 4 and 5 who had no CMV reactivation (CMV– group). Patient 6 was excluded from this analysis because the patient was positive for CMV serology but did not reactivate CMV. By day +7 after CIML NK infusion, the CMV+ group had an identifiable adaptive NK cell population (although nonadaptive NK cells still constituted the major subset), which was largely absent in the CMV– group (Figure 4A). Both the adaptive and nonadaptive NK cell clusters persisted to day +28 after CIML NK cell infusion, but nonadaptive NK cells remained the dominant subset in both the patient groups (Figure 4B). There were minimal differences in the expression of measured markers within the nonadaptive CD56^dim^ NK cell population when comparing CMV+ and CMV– patients on day +28 after CIML NK cell infusion (Figure 4C). Comparison of the CMV+ and CMV– groups with scRNA-seq also showed adaptive NK cell clusters in the infusion product (Supplemental Figure 8, C and E) that expanded on day +28 in the CMV+ patients (Figure 4D and Supplemental Table 4) and persisted to day +60 (Supplemental Figure 8, D and F). Among the CMV+ patients, the adaptive NK cell clusters on day +28 expressed more genes associated with mitochondrial ATP synthase when compared with the CMV– group (Figure 4E). Distinct clusters of nonadaptive CD56^bright^ and CD56^dim^ NK cells were also present in both the CMV+ and CMV– groups (Figure 4, D and F). The expression of genes used to characterize functionally mature CD56^dim^ NK cells such as *GZMB*, *PRF1*, and *ZEB2* (29) did not differ between the CMV+ and CMV– groups (Figure 4F and Supplemental Figure 8, E and F). Therefore, mature CD56^dim^ NK adaptive and nonadaptive NK cells expanded in both CMV+ and CMV– patients.

### Expansion of the NK cell compartment after CIML NK cell infusion is distinct from endogenous NK cells in HCT patients receiving IL-2.

**Figure 5:**
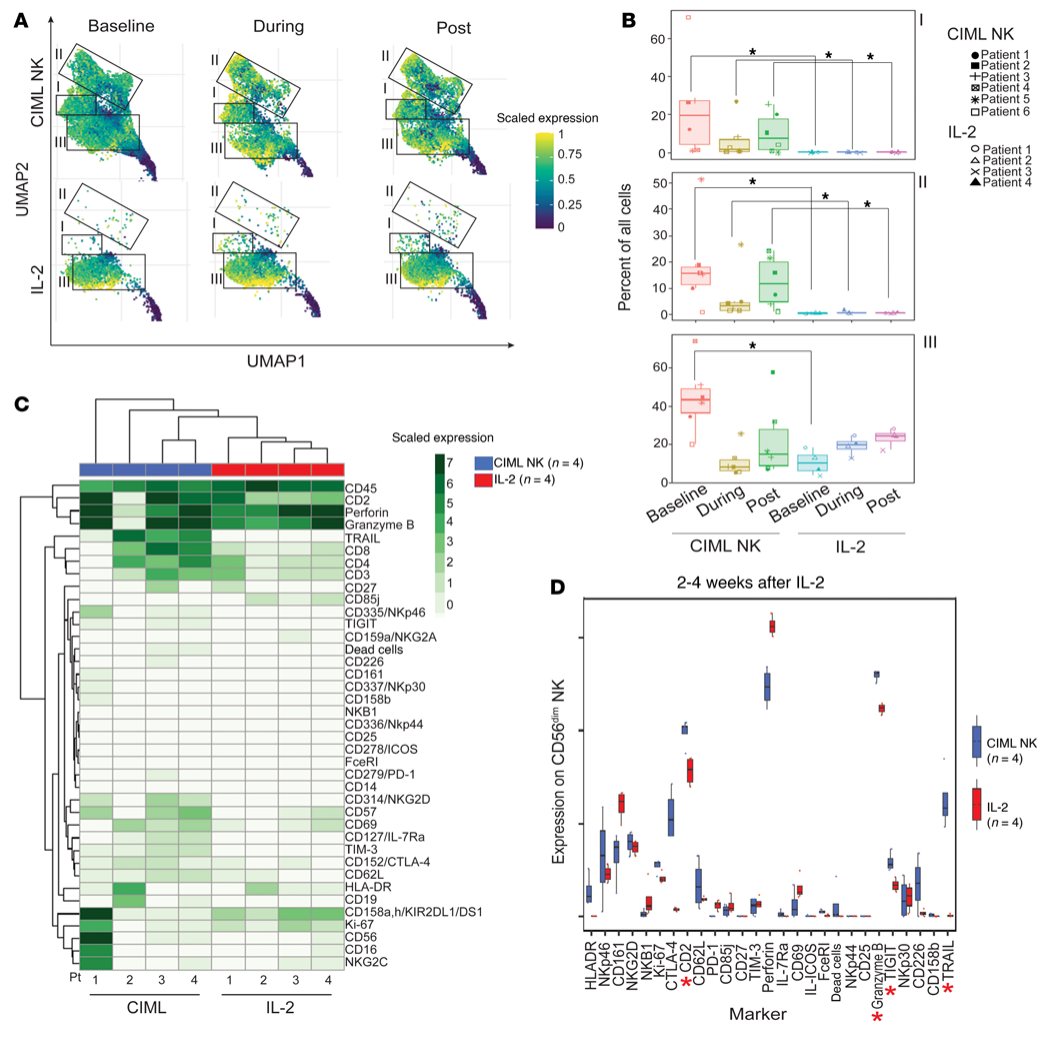
Immunophenotype of CIML NK–treated patients compared to the posttransplant IL-2–treated patients. Peripheral blood samples from 3 time points from CIML NK cell trial (*n =* 4) and IL-2 trial (*n =* 4) were evaluated using mass cytometry. The time points included at baseline prior to IL-2 start, during IL-2 treatment 1 week after starting it, and 2 to 4 weeks after completion of IL-2 therapy. (**A**) UMAP of CD3^–^CD56^+^ clusters in patients treated with IL-2 (lower insets) compared to patients on the CIML NK trial who also received IL-2 as part of the trial protocol. (**B**) Distribution of clusters of CD3^–^CD56^+^ cell subpopulations in both the CIML NK– and IL-2–treated patients. *P < 0.05 by Mann-Whitney *U* test, with significance adjusted by Holm’s method for multiple comparisons. (**C**) Distribution of marker expression on PBMCs between CIML NK– and IL-2–treated patients during therapy. (**D**) A comparison of marker expression between CIML NK trial (blue) and IL-2 trial (red) patients 2 to 4 weeks after completion of IL-2. CIML NK–treated patients exhibited a higher expression of CD2, granzyme B, TIGIT, and TRAIL compared with IL-2–treated patients. *P < 0.05 by Wilcoxon’s rank-sum test, with significance adjusted for multiple comparisons (43).

In the absence of a trackable marker on the CIML NK cells and to dissect how much expansion of the NK cell compartment was driven by IL-2 effects on endogenous post-HCT NK cells, we compared the NK cell compartment following CIML NK infusion to the corresponding NK cell compartment from 4 patients who received IL-2 after transplant on a previously published clinical trial (NCT00529035; ref. 30). On flow cytometry, CD56^dim^ and CD56^bright^ NK cell population proportions were similarly distributed in the CIML NK and IL-2 trial patients both during and 2 to 4 weeks after IL-2 therapy (Supplemental Figure 9). However, on mass cytometry, there were major differences in both NK cell subpopulation distribution and global marker expression on NK cells from the CIML NK cell and IL-2 trial samples. NK cell populations at baseline in both the CIML NK patients and in the post-HCT patients on the IL-2 trial included CD56^dim^ NK cells that were CD57^+^CD16^+^, CD57^+^CD16^–^, and CD57^–^CD16^–^ in clusters I and III and adaptive NK cells (NKG2C^+^CD57^+^) in cluster II (Figure 5A). However, clusters I and II in CIML NK cells were predominantly absent from the IL-2 trial patients at all evaluated time points (Figure 5B). Globally CIML NK cells had a higher expression of the markers CD2, TRAIL, and TIM3 on day +7 after onset of the therapy (Figure 5C). A similar comparison between the 2 patient cohorts 2 to 4 weeks after completion of their IL-2 treatment showed higher expression of CD2, TRAIL, TIGIT, and granzyme B in the CIML NK cell trial samples, suggestive of continued enhanced effector function in these patients weeks after stopping IL-2 treatment (Figure 5D).

### The NK cell compartment undergoes a phenotypic shift with expansion, but reverts back to the screening phenotype after day +60 after CIML NK infusion.

**Figure 6:**
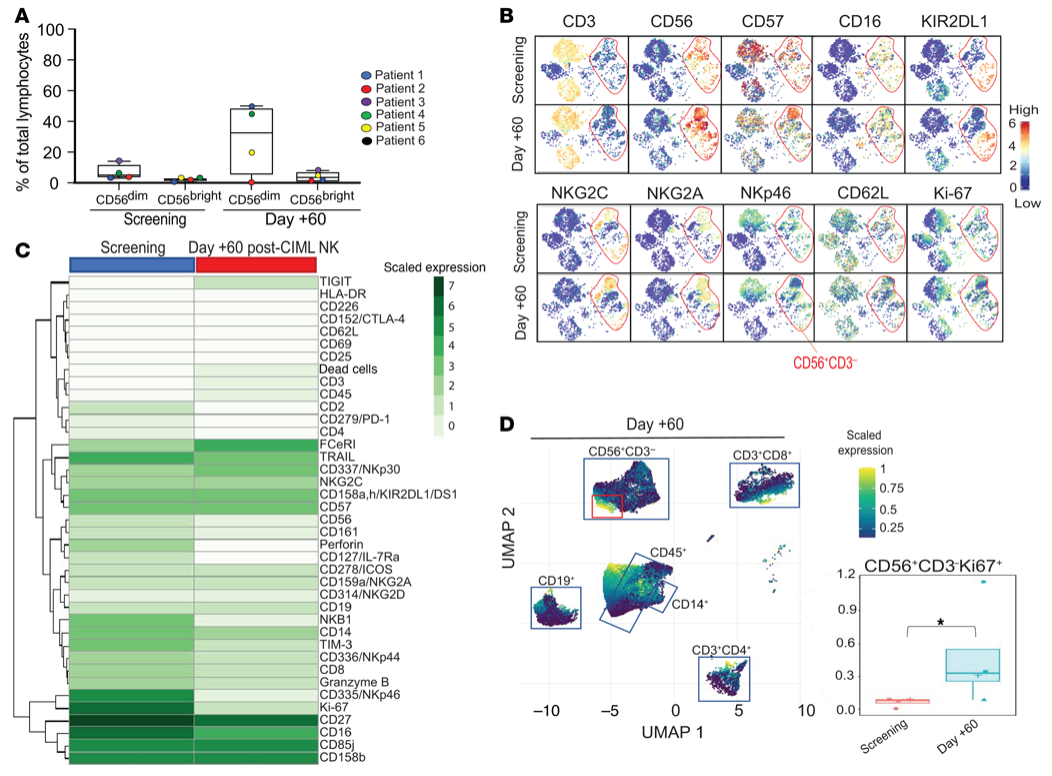
Phenotypic changes of the dominant NK cell subsets in the peripheral blood over time. NK cell phenotype was compared at screening and day +60 after CIML NK cell infusion using flow cytometry and mass cytometry. (**A**) Comparison of the CD56^dim^ and CD56^bright^ NK cell subset proportion of total lymphocyte count at screening and day +60. At screening, the median CD56^dim^ NK proportion of lymphocytes was 3.3% (min 1.3%, max 15.2%), while the median CD56^bright^ NK proportion of lymphocytes was 0.7% (min 0.1%, max 1%). On day +60, the median CD56^dim^ proportion of lymphocytes was 32.6% (min 0%, max 50.9%), while the median CD56^bright^ NK proportion of lymphocytes was 2.5% (min 0 %, max 8.4%). (**B**) tSNE plot comparison of PBMC populations at screening and day +60 time points in representative patient 3. NK cells are in the red circle. (**C**) Differential expression of mass cytometry markers in the CD3^–^CD56^dim^ cell subpopulations (Wilcoxon’s rank-sum test, with significance adjusted for multiple comparisons). Screening (*n =* 6) and day +60 (*n =* 5). Patient 5 did not have a day +60 sample. (**D**) Clustering of PBMCs to identify the Ki67^+^ subpopulation (red squares) of CD3^–^CD56^+^ NK cells. CD3^–^CD56^+^Ki67^+^ cells as a proportion of total PBMCs at both screening and day +60. Individual patient values are shown superimposed on the box plots. The median percentage of CD3^–^CD56^+^Ki67^+^ cells at screening and day +60 was 0.075% (min 0%, max 0.09%) and 0.32% (min 0.07%, max 1.15%), respectively. *Differential abundance, with *P <* 0.05 using Wilcoxon’s rank-sum test.

CD56^dim^ NK cells accounted for approximately 35% of all lymphocytes in the peripheral blood at the day +60 time point, in contrast to a median of 7% at the screening time point (Figure 6A). CD56^dim^ NK cells by day +60 phenotypically resembled the corresponding NK cells at screening (Figure 6B). There were no significant differences in mass cytometry marker expression between the 2 time points (Figure 6C). We sought to identify the subpopulation of NK cells that might sustain this greater abundance by identifying a cluster among the NK cells that was highly positive for Ki67. The relative abundance of this subpopulation on day +60 was significantly increased compared with the screening time point in all evaluable patients (0.32% vs. 0.075% of all PBMCs, *P =* 0.04 by Wilcoxon’s rank-sum test), although the number of Ki67^+^ NK cells was less than 1% of PBMCs at both time points (Figure 6D). Similar to the predominant CD56^dim^ population, there were no mass cytometry markers that were significantly differentially expressed among the CD56^bright^ NK cells between the 2 time points (Supplemental Figure 10).

### NK cells traffic to sites of disease after CIML NK cell infusion.

**Figure 7:**
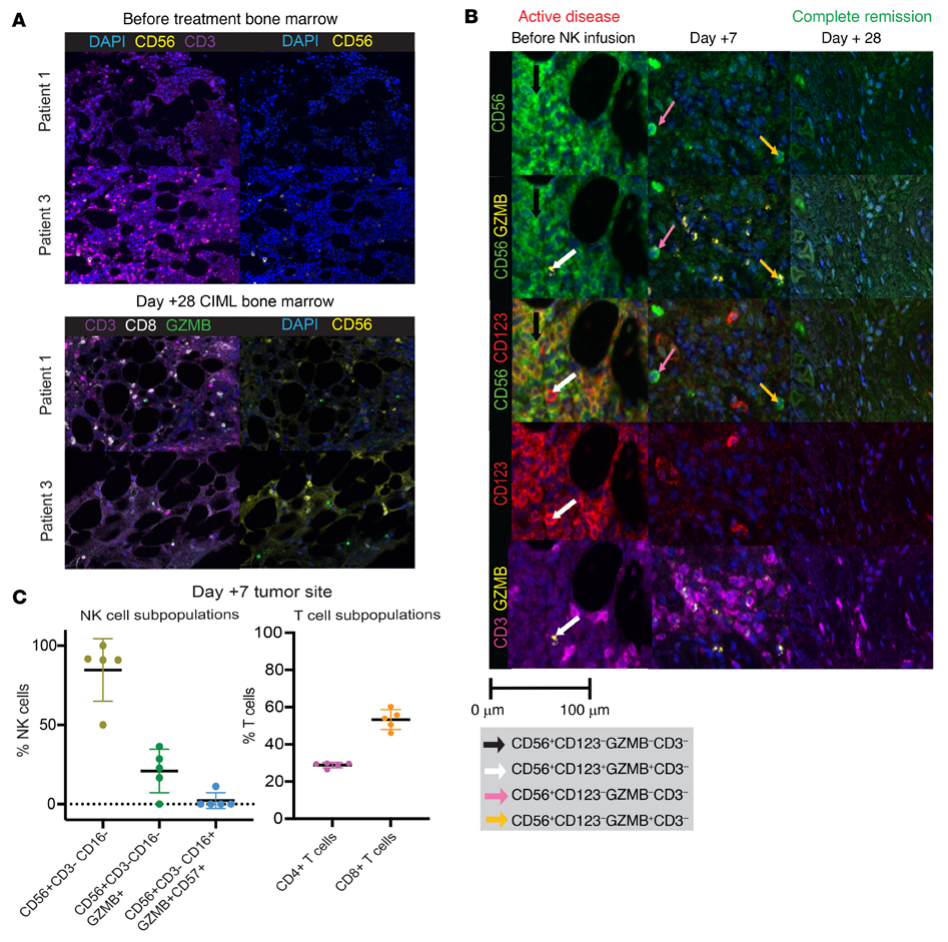
Immune effector cell infiltration at the site of disease. (**A**) Multiparameter immunofluorescence imaging (MIFI) was applied to the pretreatment bone marrow and day +28 fter CIML NK therapy applied to 2 representative patients. NK cell infiltration was relatively sparse in patients 1 and 3 before therapy, as exemplified by the general lack of CD56 positivity relative to the predominance of CD3 positivity. In contrast, on day +28 after CIML NK infusion, the CD3^–^CD56^+^ staining was relatively enhanced. (**B**) Infiltration of immune effector cells in the patient with BPDCN in the context of active CD123^+^ extramedullary disease. Prior to CIML NK cell infusion, diseased cells (white arrow) were juxtaposed to some NK cells (black arrow) and CD3^+^ cells. On day +7 after CIML NK infusion, substantial reduction in the CD123^+^ blasts, with residual NK cell subpopulations (pink and yellow arrows) and CD3^+^ cell populations noted. By day +28 after CIML NK infusion, the extramedullary lesion was involuted and with no detectable blasts and NK or T cell cellular infiltrate. Original magnification, ×200 (**A** and **B**). (**C**) Quantitation of NK cell and T cell subpopulations (individual dots represent quantitation within a specific field in the biopsy, 5 fields evaluated) from day +7 MIFI panel from extramedullary disease site in BPDCN patient.

We evaluated whether expansion of the NK cell populations after CIML NK cell infusion is associated with the presence of NK cells at sites of disease. Multiparameter immunofluorescence imaging (MIFI) was used to investigate the expression of multiple markers in the bone marrow and tissue biopsy samples before and after CIML NK cell infusion. Patient 1 had nearly no detectable NK cells in the bone marrow on day +100 after HCT, and yet had a definite NK cell infiltration on day +28 after CIML NK cell infusion despite the marrow being hypocellular (<10% cellularity) (Figure 7A). Patient 3, on the other hand, had NK cells present in the day +100 post-HCT bone marrow (50% cellularity) and in the day +28 post–CIML NK infusion bone marrow (Figure 7A). Further longitudinal evaluation of available bone marrow samples from patient 3 showed a paucity of NK cells on day +60 and at the time of relapse after CIML NK cell infusion. In patient 4 with extramedullary disease, CIML NK cell infusion was associated with an NK cell infiltrate at the tumor site 7 days after infusion. This infiltrate was juxtaposed to the CD123^+^ leukemia blasts, which were substantially reduced on day +7. The leukemia blasts and NK cells were undetectable by day +28 after CIML NK cell infusion (Figure 7B). The reduction in blast burden in this patient was also associated with an influx of T cells into the tumor site. Further characterization of the immune cell infiltrate in this patient on day +7 revealed that most NK cells were CD16^–^, with some cells expressing granzyme B and only a minority expressing CD57 (Figure 7C). Similarly, the majority of the T cells were CD8^+^. In all other patients with bone marrow involvement of their disease, NK cells were present in the marrow on day +28 after infusion (Supplemental Figure 11).

### AML blasts present at relapse after CIML NK therapy express inhibitory NK cell ligand genes.

**Figure 8:**
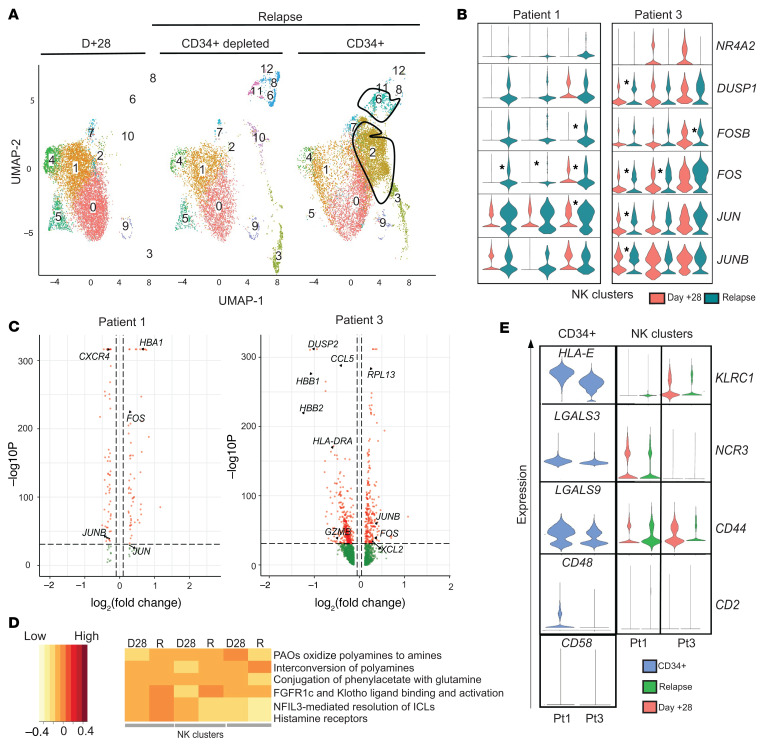
Transcriptomic evaluation of NK cells and AML blasts at the time of disease relapse by scRNA-seq. (**A**) Representative UMAP from scRNA-seq analysis in patient 1. The outlined populations are CD34^+^ blasts that were identified at relapse. (**B**) Violin plots corresponding to the expression of activation markers in CD3^–^CD56^+^ NK cell clusters defined using scRNA-seq. *P < 0.05 by Wilcoxon’s rank-sum test, with significance adjusted for multiple comparisons (50). (**C**) Volcano plots for patient 1 (left) and patient 3 (right) comparing gene expression in NK cell–containing scRNA-seq clusters at day +28 to relapse. Genes on the left of the violin plot are downregulated while those on the right are upregulated at the time of relapse. (**D**) Gene set enrichment analysis corresponding to differentially expressed genes in NK cell clusters derived from scRNA-seq data in patient 1 as a representative example. Within every cluster, every pair of columns corresponds to day +28 (D28) and relapse (R) time points. The intensity of expression is represented by the color of the legend on the left. (**E**) Expression of specific NK cell receptors within the NK clusters on day +28 and relapse in patient 1 (P1) and patient 3 (P3) and their corresponding NK ligands on the relapsed leukemia blasts are indicated in blue (CD34^+^ cells).

Patients 1 and 3 both exhibited long-term persistence of NK cell populations that were present in their infusion products. At the time of AML relapse, they both had a coexistence of leukemia blasts with the NK cells in the peripheral blood, providing an opportunity to potentially understand NK and leukemia cell interactions (Figure 8A). Using scRNA-seq, we conducted a differential expression analysis comparing NK cell clusters between day +28 and relapse time points in both patients 1 and 3 to determine whether there was a change associated with relapse (Figure 8B). All NK cell clusters at the time of relapse expressed genes associated with NK cell activation (Figure 8B). The *FOS* gene had increased expression at the time of relapse in both these patients (Figure 8C and Supplemental Tables 5 and 6). All the NK clusters in patient 1 had a significant reduction in the expression of *CXCR4* at the time of relapse (Figure 8C). Patient 3, on the other hand, had an upregulation of genes associated with the CD56^bright^ phenotype of NK cells at the time of relapse (Supplemental Table 6). We conducted gene set enrichment analysis on all differentially expressed genes in the CD56^dim^ and CD56^bright^ NK cell clusters that were present at relapse in patients 1 and 3 (Figure 8D). We identified genes in the *FGFR1c* pathway, thought to be involved in the differentiation of CD56^bright^ NK cells to a CD56^dim^ phenotype (31), as being differentially expressed at the time of AML relapse compared with day +28 in both patients. Other pathways that exhibited differential expression included those involving polyamines and histamine receptors.

We next evaluated whether post–CIML NK relapse of AML was associated with the expression of ligands for activating and inhibitory receptors present on the persistent NK cells in the peripheral blood. Using scRNA-seq, we identified CD34^+^-containing clusters enriched for AML blasts, and confirmed that these did not express typical NK cell markers (Supplemental Figure 12). *HLA-E*, an inhibitory ligand that binds NKG2A and CD94 (*KLRD1*), was highly expressed on the CD34^+^ clusters in patient 1 but its expression was lower on the CD34^+^ clusters in patient 3 (Figure 8E). The leukemia-containing clusters were found to have a low expression of *CD48* and *CD58*, the genes coding for activating ligands that bind CD2. Galectin-9 (*LGALS9*), a known inhibitory ligand for NK cells, was expressed predominantly on the CD34^+^ clusters in both patients. TIM3 (*HAVCR2*), a receptor for galectin-9, was not expressed in the CD34^–^ clusters at either the expansion or relapse time point, but its receptor, CD44, was expressed at relapse. The expression of most other NK activating and inhibitory ligands was either not present or not assessable with 3′ scRNA-seq (Supplemental Figure 13).

Since CD2 ligands were found to be downregulated in the context of AML relapse and CD2 was highly expressed on CD56^dim^ CIML NK cells (Figure 5D), we evaluated the effect of blocking CD2 on CIML NK activation, cytokine secretion, or cytotoxicity in vitro using a blocking anti-CD2 monoclonal antibody. While blockade of CD2 led to reduced secretion of TNF-α in conventional NK cells, it did not have any significant effect on CIML NK cell activation, cytokine secretion, or cytotoxicity (Supplemental Figure 14), suggesting that other activating NK cell receptors are able to compensate for lack of CD2 signaling for activation in CIML NK cells.

## Discussion

We demonstrate that infusion of donor-derived CIML NK cells into an immune-compatible environment for the management of post–haplo-HCT relapse of myeloid disease is safe and results in rapid expansion and long-term persistence of NK cells. The expansion of the NK cell compartment following CIML NK cell infusion was distinct from IL-2 effects on endogenous posttransplant NK cells and was not dependent on CMV reactivation. In preliminary outcomes of this ongoing trial, infusion of CIML NK cells was associated with a reduction in measurable disease burden in 4 of 6 treated patients, including a patient with BPDCN. Response to therapy was sustained for several months. CIML NK cells were well tolerated, with the main adverse effect being the development of cytopenia in most patients. Importantly, infusion of these donor lymphocytes was not associated with severe CRS or neurotoxicity. Furthermore, in spite of the administration of low-dose IL-2 in the posttransplant setting, no patient developed any GvHD.

The lifespan of conventional NK cells is thought to be in the range of 12 to 14 days, but adoptive transfer of CIML NK cells in a syngeneic murine model was associated with significant prolongation of their expected lifespan (23). In a previous clinical trial using CIML NK cells to treat relapsed/refractory AML in a nontransplant HLA-mismatched setting, the donor-derived NK cells could not be detected beyond day +28 after infusion (21). In the current clinical trial, expansion of donor CIML NK cells and subsequent persistence of these cells for several months was noted. We hypothesize that this was due in part to the immune-compatible posttransplant milieu since all treated patients had high T cell chimerism at the time of CIML NK infusion.

CMV reactivation likely contributed to the expansion and persistence of immunophenotypically and transcriptionally identified adaptive CD56^dim^ NK cell subsets. However, nonadaptive CD56^dim^ NK cells were the dominant subset and expanded in both the CMV+ and CMV– patients. Furthermore, the presence of CMV did not significantly affect the surface marker or gene expression of nonadaptive CD56^dim^ NK cells. This suggests that CMV reactivation was not the sole driver of NK cell expansion. Additional work will be required to determine whether there is any difference in antitumor activity between the adaptive NK cells expanded in response to CMV and the nonadaptive NK cells.

The expanded NK cell compartment following infusion of CIML NK cells was associated with presence of NK cells at disease sites and reduction of disease burden. This was most dramatic in patient 4 with BPDCN whose disease burden visibly shrank and became undetectable on PET-CT. MIFI demonstrated the presence of NK cell and CD8^+^ T cell infiltrates in the extramedullary mass in this patient, suggesting a possible interaction between these 2 cell populations in mediating disease reduction and preventing disease relapse. The infiltration of CD8^+^ T cells into the site of disease was also seen in patient 3. Further work is required to explore the possible interaction between CD8^+^ T cells that appear to infiltrate disease sites following CIML NK infusion and the infused NK cell subsets.

While the use of CIML NK cells was safe and not associated with severe CRS, neurotoxicity, or GvHD, it is of note that all patients with bone marrow involvement of myeloid disease exhibited pancytopenia. In 2 of these patients, a CD34^+^-selected stem cell boost was required to promote peripheral blood cell recovery, while in the others the peripheral blood counts recovered on their own. Prolonged pancytopenia after adoptive cell transfer was observed in prior CAR-T trials and may be related to the lymphodepleting chemotherapy (32). One patient exhibited clinical and laboratory features of grade 2 CRS, requiring treatment with tocilizumab that resolved the syndrome. CRS was reported in a recently published haploidentical NK cell therapy trial, although the higher reported rate in that trial could have been due to the use of subcutaneous IL-15 to expand the NK cells (33). Consistent with this trial and other published NK cell trials, no patient developed any GvHD despite receiving a product that is mismatched to the recipient. The tolerability of the adoptively transferred CIML NK cell therapy in this trial is similar to what has been seen in other NK cell therapy trials, including those using IL-21–expanded NK cells (34, 35).

The deeper immunophenotypic evaluation of infused CIML NK cells using mass cytometry revealed interpatient heterogeneity, likely reflecting the diversity of the NK cell populations inherent in the donor (36). It was demonstrated that the mass cytometry–defined CIML phenotype persists long term as an imprint superimposed on the original NK cell repertoire. This imprint was not driven by IL-2 alone, as a comparator group of patients who received the same dose of IL-2 at least 6 months after transplant did not yield expansion of the same CD56^dim^ NK cell subpopulations. The NK cell compartment following CIML NK infusion expressed higher levels of CD2, TIGIT, TRAIL, and granzyme B that persisted several weeks after the last dose of IL-2, suggesting that this set of markers could perhaps distinguish the CIML NK–expanded NK cells from endogenous NK cells. We hope to build upon this finding in future studies with larger patient numbers, as lack of distinct marker(s) makes tracking CIML NK cells in vivo challenging particularly when using autologous products and in a post–allo-HCT setting. By day +60 after infusion, the NK cell compartment largely resembled that seen prior to infusion of CIML NK cells.

scRNA-seq analysis of the NK cell compartment at the time of AML relapse after CIML NK cell therapy demonstrated the persistence of both CD56^bright^ and adaptive NK cell populations, among others. Gene set enrichment analysis suggested that in the patient with a sizeable expansion and persistence of CD56^bright^ NK cells, there was downregulation of the *FGFR1c* pathway involved in NK cell maturation from CD56^bright^ to CD56^dim^, potentially explaining why the CD56^bright^ NK cells were able to persist (31). The expression of HLA-E in the relapsed AML blasts in this patient with an expanded NKG2A-expressing CD56^bright^ NK population suggests a potential evasion mechanism, in agreement with previous analysis of residual leukemic cells following allogeneic NK cell therapy (37). Since this patient also exhibited an infiltrate of CD8^+^ T cells at the site of disease, possibly promoted by the cytokine secretion from the CD56^bright^ NK cells, evasion of CD8^+^ T cells may have contributed significantly to the relapse mechanism.

Additional mechanisms of AML relapse after CIML NK therapy may be associated with the absence of ligands for CD2, a receptor that is expressed on the NK cell populations after infusion, and that is involved in mediating tumor target cytotoxicity via interaction with CD16 (38). In patient 1, in whom most of the expanded and persistent NK cells exhibited a mature cytotoxic CD56^dim^CD16^+^ phenotype, the absence of expression of CD48 and CD58 on relapsed leukemia could suggest a resistance mechanism. Our in vitro analysis, however, suggests that CD2 blockade is not sufficient to reduce CIML NK cell activation, cytokine secretion, or cytotoxicity. This does not exclude CD2 as a relevant receptor for targeting of myeloid disease, as conventional NK cells were affected by the blocking antibody, but does suggest that the lower activation threshold of CIML NK cells leads to additional mechanisms being involved in mediating escape of AML from their effects. Such additional mechanisms of AML relapse may involve the inhibitory ligand galcetin-9, since its receptor, CD44 (39), was expressed on the CD34^–^ PBMCs at the time of relapse. The expression of galectin-9 may have played an additional role in the survival of residual leukemia clones early after NK cell infusion when its receptor TIM3 was more highly expressed in the infused CIML NK cell product.

While this study consistently demonstrated the rapid expansion and persistence of donor-derived CIML NK cells without any GvHD or severe CRS complications, it has important limitations. First, the number of treated patients was relatively small. Furthermore, as the infused NK cells shared HLA expression with the NK cells already present in the transplanted patient and there was no traceable marker in the infused CIML NK cells, it was not possible to use donor chimerism or other direct means to track the infused NK cells. The mass cytometry marker signature determined by comparison of the NK compartments from the CIML NK trial patients and IL-2–treated posttransplant patients must also be interpreted with caution, as the NK cells being compared came from patients who are not at identical times in their posttransplant course. Although these limitations prevent immediate generalizations of the findings, the deep high-throughput analyses of longitudinally collected samples do reveal a number of important hypothesis-generating observations with the potential for clinical translation in the near future. Additional work is necessary to elucidate the mechanism(s) of the prolonged alterations in the NK cell compartment after donor CIML NK cell infusion, as well as their interaction with other immune effector cells as a means to treat posttransplant relapse of myeloid disease.

### Conclusion

In summary, we demonstrate that CIML NK cells infused into an immune-compatible milieu in the context of a haploidentical stem cell transplant are associated with marked NK expansion and long-term persistence. We show that CIML NK cell products comprise predominantly cytotoxic CD56^dim^ NK cells but also exhibit donor-specific heterogeneity in both the presence of additional NK cell subsets as well as in the expression of NK cell markers associated with the memory-like phenotype. The NK cell subpopulations present at the time of NK cell infusion undergo significant phenotypic changes associated with enhanced NK cell activity and the development of a memory-like phenotype by day +28, but by about day +60 after infusion phenotypically resemble the NK cells present prior to infusion in significantly increased numbers. We also show that expansion of the NK cell compartment following CIML NK cell infusion is distinct from IL-2 effects on endogenous posttransplant NK cells and is not dependent on CMV reactivation. Finally, we speculate that relapse of AML after CIML NK cell therapy is associated with expression of NK inhibitory ligands, and may occur in the context of evasion of CD2-mediated activation in addition to other mechanisms that remain to be determined. Because of their capacity for rapid expansion and long-term persistence, CIML NK cells offer an attractive platform for further improvement of NK cell therapies by combining them with immunomodulatory agents and/or by arming them with CAR gene constructs.

## Methods

### Study design.

Patients with relapsed myeloid disease, including AML, MDS, myeloproliferative neoplasm (MPN), or BPDCN following haplo-HCT were eligible to be treated in an ongoing single-center phase I dose de-escalation clinical trial of CIML NK cell therapy (NCT04024761). The primary endpoints are safety and identification of the maximum tolerated dose (MTD) of CIML NK cells administered following stem cell transplantation. The trial schema involves treatment of 5 patients at a starting dose of 5 × 10^6^ to 10 × 10^6^ cells/kg, a second dose level of 1 × 10^6^ to 2 × 10^6^ cells/kg in the event of at least 2 dose-limiting toxicities at the starting dose, and plan to treat 10 additional patients at the MTD in phase Ib. CIML NK cells were derived from the same donor who provided stem cells for the original transplant. Patients receiving therapy had relapsed disease that was confirmed by morphological evaluation, flow cytometry, next-generation sequencing, or PET-CT imaging, did not previously receive DLI, and had to have at least 20% donor T cell chimerism. NK cells were purified from nonmobilized apheresis products by a 2-step CD3 depletion followed by CD56^+^ selection (CliniMACS device, Miltenyi Biotec), consistently achieving greater than 90% purity for CD3^–^CD56^+^ NK cells. The purified NK cells were subsequently activated overnight (12–16 hours) with IL-12 (10 ng/mL), IL-15 (50 ng/mL), and IL-18 (50 ng/mL) to generate the CIML NK cells under GMP conditions. The cells were washed and were subject to quality control assessment, including a requirement for a T cell dose of less than 3 × 10^5^ cells/kg in the product. All patients treated so far were infused as inpatients by their healthcare team at the starting NK cell dose of 5 × 10^6^ to 10 × 10^6^ cells/kg over at least 15–30 minutes after a course of lymphodepleting chemotherapy. Patients 1, 3, and 4 were treated with a combination of fludarabine (25 mg/m^2^, days –6, –5, –4, –3, and –2), and cyclophosphamide (60 mg/kg, given on days –5 and –4). Patient 2 received a protocol deviation and was treated at a reduced dose of fludarabine (30 mg/m^2^ i.v. over 1 hour on days –4, –3, and –2) and cyclophosphamide 60 mg/kg i.v. over 1 hour (on days –4 and –3) due to concern about tolerating potential chemotherapy-induced cytopenia. The lymphodepleting chemotherapy doses for patients 5 and 6 were adjusted to fludarabine (30 mg/m^2^ i.v. over 1 hour on days –5, –4, and –3) and cyclophosphamide 500 mg/m^2^ i.v. over 1 hour (on days –5 and –4) after a trial amendment. The first 5 patients were treated with a low dose of human recombinant IL-2 (1 × 10^6^ IU/m^2^) subcutaneously every other day for a total of 7 doses. Patient 6 received only 2 doses of IL-2 due to the development of transaminitis. Response evaluation included day +28 bone marrow biopsy and a PET-CT in patient with extramedullary disease (patient with BPDCN). All patients were followed for up to 5 years or until disease relapse or mortality from the time of trial recruitment as per protocol.

### Patient and healthy donor samples.

Patients with relapsed AML, MDS, MDS/MPN, or BPDCN provided written informed consent prior to participation under an IRB-approved protocol at the Dana-Farber Cancer Institute (DFCI) where all protocol procedures were performed and data were collected. Correlative samples included peripheral blood collected at screening for the trial, on day –7 (before starting lymphodepletion), day 0, day +7, day +21–28, day +42, day +60, day +100, and relapse time points after infusion. Healthy donor PBMCs were isolated by Ficoll centrifugation, and NK cells were purified using RosetteSep (STEMCELL Technologies). All PBMCs were cryopreserved according to a standard internal laboratory protocol at the time of collection from patients.

Cryopreserved PBMC samples from 4 additional patients were retrieved and analyzed from a previously published clinical trial (30) involving subcutaneous administration of IL-2 (at the same dose as in the current CIML NK trial) in the post–stem cell transplant setting (NCT00529035). These samples included baseline (before IL-2), during (7 days after initiation of IL-2), and after (2–4 weeks after completion of IL-2). NK cells from these patients were analyzed using flow cytometry and mass cytometry and their phenotype compared to the NK cells from our CIML NK trial patients. These analyses were performed to potentially evaluate major phenotypic changes with the use of IL-2 on endogenous NK cells in the post-HCT setting versus CIML NK cells in the current study.

### Flow cytometric analysis.

A custom NK and T cell panel of antibodies was used (Supplemental Tables 7 and 8). All cell staining for flow cytometry was performed as previously described (21), and data were acquired on a BD LSR Fortessa flow cytometer and analyzed using FlowJo (Tree Star) software. Gating strategies are described in Supplemental Figure 3. The absolute lymphocyte count was measured using a clinical assay. For any lymphocyte population not measurable directly with the clinical assay, the absolute count of this population was determined by multiplying its percentage of total lymphocytes, as measured with flow cytometry, by the total lymphocyte count measured at the same time point using the clinical assay.

### Mass cytometry (CYTOF).

Metal-tagged antibodies used for our mass cytometry panel are listed in Supplemental Table 9. In-house conjugations of antibodies were performed with a Maxpar labeling kit per the manufacturer’s instructions (Fluidigm). All antibodies were used per the manufacturer’s recommendation (Fluidigm).

Cryopreserved patient PBMC samples were thawed, counted using acridine orange and propidium iodide (AO/PI), and pelleted by centrifugation at 800*g* for 3 minutes. Cells were then incubated in 103Rh viability stain for 15 minutes, washed in CyFACS, and incubated with undiluted Human TruStain FcX for 10 minutes for Fc receptor blocking. Antibody master mix was applied to cell suspensions for 30 minutes, washed and fixed/permeabilized with FoxP3 Fixation/Permeabilization Concentrate and Diluent, prepared following the manufacturer’s guidelines (eBioscience). A mix of intracellular antibodies prepared with 1× Perm Wash was added to each sample and incubated for 30 minutes. Next, cells were washed with 1× Perm Wash and incubated overnight at 4°C in FoxP3 Fixation/Permeabilization Concentrate and Diluent, containing 191/193Ir DNA intercalator (Fluidigm). Prior to acquisition, samples were transferred to 5 mL round-bottom polystyrene tubes with cell strainer caps, washed and filtered with Cell Staining Buffer (CSB), Cell Acquisition Solution (CAS), and resuspended in CAS supplemented with EQ Four Element Calibration Beads (1:10) (Fluidigm).

All mass cytometry data were collected on a Helios Mass Cytometer (Fluidigm). The instrument was tuned using CYTOF Tuning Solution according to the Helios User Guide (Fluidigm, pp. 60–68). A brief overview of tuning steps includes Pre-XY Optimization, Mass Calibration, XY Optimization, DV Calibration, Dual Calibration, Gases/Current Calibration, and QC report. EQ Four Element Calibration Beads (1:10 in CAS) were used according to the manufacturer protocol before and during acquisition. The data were normalized using the FCS Processing tab of Fluidigm CYTOF software v7.0.8493.

Data analysis was manually performed using FlowJo 10.7.1. Initial data cleanup was based on a previously described gating strategy (40). Cell events were gated to remove dead cells and debris through biaxial plots of time versus event length, beads (for removal of the EQ Calibration Beads), and Gaussian-derived parameters (residual, width, offset). The viability stain 103Rh was used to gate out dead cells on PBMC populations. All viable cells were back-gated on both DNA parameters (191Ir and 193Ir) to ensure no doublets were included. Clustering of NK cell populations was performed both with R-Phenograph and FlowSOM methods in R (41–44).

### Luminex cytokine assays.

Patient plasma samples were thawed and prepared for soluble analyte assay according to previously published methods (45, 46). Analytes were measured on a Luminex FLEXMAP3D per the manufacturer’s protocol. Soluble eotaxin, GM-CSF, GROα, IFN-α, IFN-γ, IL-1β, IL-1α, IL-1RA, IL-2, IL-4, IL-5, IL-6, IL-7, IL-8, IL-9, IL-10, IL-12 p70, IL-13, IL-15, IL-17A, IL-18, IL-21, IL-22, IL-23, IL-27, IL-31, IP-10, MCP-1, MIP-1α, MIP-1β, RANTES, SDF1α, TNF-α, and TNF-β were tested. Of all soluble markers measured, 33 were within detectable range and could be quantified by extrapolation of mean fluorescence intensities to the respective standard curve between lower limit of quantitation and upper limit of quantitation. Analyte concentration for each patient was calculated using standard curves. Fold changes were calculated as a ratio relative to the patient’s baseline (C0D1) (47–49).

### Functional and flow-based cytotoxicity assays.

Frozen PBMCs were thawed and cultured overnight in RP-10 medium (RPMI 1640 supplemented with 10% FBS, 1× penicillin/streptomycin, 2 mM L-glutamine, and 7.5 mM HEPES) with 1 ng/mL IL-15. K562 cells (ATCC CCL-243) were cultured in RP-10 medium and labeled with 5 μM CellTrace Violet (Thermo Fisher Scientific) in PBS for 20 minutes at 37°C. PBMCs and K562 cells were washed twice with RP-10 and cocultured at the indicated effector/target (E:T) ratios. To measure NK cell cytotoxicity, PBMCs and K562 cells were cocultured for 4 hours, and then stained with PE–annexin V and 7-AAD (BD Biosciences) for 15 minutes at room temperature. To measure intracellular IFN-γ and degranulation, PBMCs and K562 cells were cocultured for 1 hour, followed by the addition of 0.2 μL GolgiPlug (BD Biosciences), 0.13 μL GolgiStop (BD Biosciences), and 1 μL of APC-CD107a (BioLegend). After an additional 5 hours of coculture, cells were stained for intracellular IFN-γ using Cytofix/Cytoperm (BD Biosciences). Cells were acquired using BD LSR Fortessa and analyzed using FlowJo.

### scRNA-seq and analysis.

Cryopreserved PBMC samples from patients were thawed and diluted with prewarmed complete RPMI 1640, and cells were centrifuged at 200*g* for 10 minutes to remove dead cells. Cell pellets were resuspended in 25% Percoll solution in PBS and centrifuged at 300*g* for 10 minutes to further remove dead cells. Live cells were resuspended in 2% FBS/PBS for single-cell isolation and cDNA library construction. The relapse samples were depleted of CD34^+^ cells using a CD34^+^ positive selection kit (STEMCELL Technologies), with the CD34^+^ cells resuspended as independent tumor samples.

Single-cell libraries were prepared using the 10× Chromium Next GEM Single Cell 3′ Kit (10× Genomics), according to the manufacturer’s instructions. The single-cell cDNA libraries were sequenced by NovaSeq S4 flowcell (Illumina), with data deposited in the NCBI Gene Expression Omnibus database (GEO GSE198141). Raw sequences were demultiplexed, aligned, and filtered. Barcode counting and unique molecular identifier (UMI) counting was done with Cell Ranger software v3.1 (10× Genomics) to digitalize the expression of each gene for each cell. Analysis was performed using the Seurat 3.0 package (50). Data from individual samples were analyzed separately prior to combining the data from multiple samples. The outlier cells with very low number of gene features (<500) or low total UMI (<1000), or high number of gene features (>5000) or high total UMI as doublets (>20,000) were removed. Cells with high mitochondrial ratio (>15%) from each data set were also removed. Subsequently, samples were combined based on the identified anchors for the following integrated analysis. Principal component analysis (PCA) was performed and the first 15 principal components (PCs) were used to cluster cells using the Louvain algorithm in Seurat. These PCs were then used to run UMAP clustering. Well-defined marker genes for each cluster were used to identify potential cell populations, such as T cells (*CD3E*, *CD4*, *CD8A*), naive T cells (*IL7R*, *CCR7*), B cells (*CD19*, *CD20*, *SDC1*, *MS4A1*), CD14^+^ monocytes (*CD14*, *LYZ*), NK cells (*NKG7*, *GNLY*), and platelets (*PPBP*). Differential gene expression analysis was performed in Seurat using the nonparametric Wilcoxon’s rank-sum test [Find markers () function] and the MAST test. Volcano plots were used to visualize data and were generated in the ggplot2 package in R (https://cran.r-project.org/package=ggplot2). For gene sets representing specific cellular functions or pathways, we performed functional enrichment analysis with the online tool Reactome GSA (https://www.biorxiv.org/content/early/2020/04/18/2020.04.16.044958) (51).

### Statistics.

All flow cytometry data were tested for normal distribution (Shapiro-Wilk test), and if the data were not normally distributed, the appropriate nonparametric tests were used (GraphPad Prism v5.0). *P* values corresponding to the differential expression using mass cytometry data were acquired using the diffcyt package in R (52). Differential expression of scRNA-seq data was performed using Wilcoxon’s rank-sum test as part of the Seurat package (50). All statistical comparisons are indicated in the figure legends. All comparisons used a 2-sided α of 0.05 for significance testing, with adjustment for multiple comparisons where required.

### Study approval.

This study was reviewed and approved by the institutional review board of the DFCI, Boston, Massachusetts, USA (ClinicalTrials.gov NCT04024761). The study was performed in compliance with the provisions of the Declaration of Helsinki and Good Clinical Practice guidelines. Written informed consent was obtained from participants before inclusion in the study.

## Author contributions

RMS and GCB contributed equally to the study, with RMS listed first because he is the principal investigator on the trial. RMS, SN, RJS, JR, and RR designed the clinical study protocol. RMS, GCB, and RR designed the correlative studies. SN prepared CIML NK products under GMP guidelines and was responsible for quality control of the products. CR and MSH processed and prepared patient samples for correlative studies. JVC, AKA, MT, and GCB performed the NK cell functional assays. BR and YA performed flow cytometry. GH performed single-cell RNA-seq. JB performed mass cytometry. SJR and NC performed multiparameter immunofluorescence on bone marrow biopsies. MS and YZA assisted with the design and conduct of the CD2 blockade functional experiments. YA assisted with conducting and analyzing flow cytometry on patient samples. JOW and KLP conducted the MIFI experiment on tissue biopsies. AAL provided BPDCN patient samples. CSC, JHA, VTH, JK, and MG recruited clinical trial participants. RCL and HTK reviewed the data from correlative studies and statistical analyses, respectively, and provided critical feedback. RMS and GCB analyzed all the data and designed the figures. RMS, GCB, and RR interpreted the data and wrote the first draft of the manuscript. KJM, CJW, JC, RJS, and JR reviewed the manuscript, and provided critical feedback on the results of the correlative studies. All authors revised the manuscript critically and approved the final version.

## Supplementary Material

Supplemental data

ICMJE disclosure forms

## Version 1 03/29/2022

In-Press Preview

## Version 2 06/01/2022

Electronic publication
