# Supplementary material for: Expansion, persistence, and efficacy of donor memory-like NK cells infused for posttransplant relapse
Source: J Clin Invest. 2022 Jun 1;132(11):e154334. doi: 10.1172/JCI154334 (PMC9151697; doi:10.1172/JCI154334)
Supplement: Supplemental data [file jci-132-154334-s019.pdf]

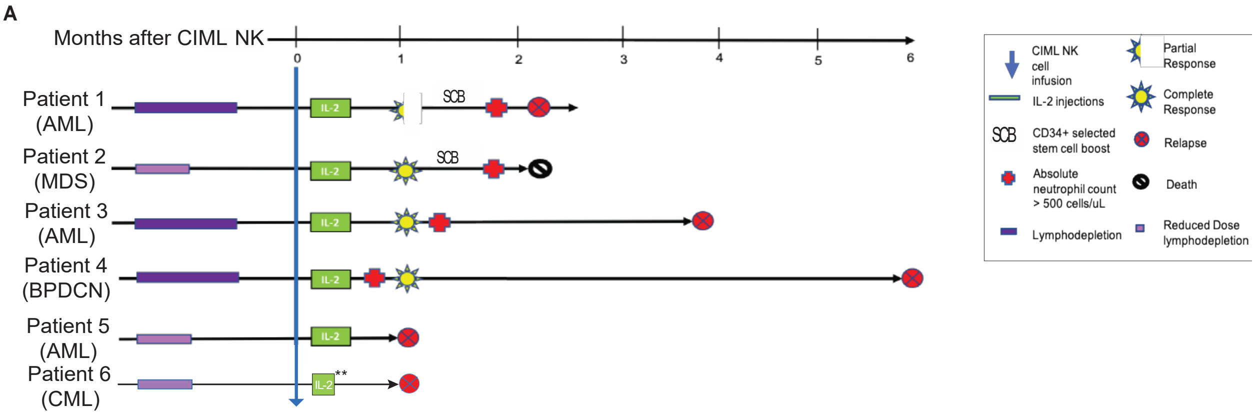

Figure S1. **Clinical course and responses of patients treated with CIML NK cell therapy. A** The clinical outcomes of patients treated with CIML NK cells are shown. All patients had measurable disease post haplo-HCT and prior to receiving CIML NK cells. Complete response and morphologic leukemia-free state are defined per the ELN 2017 criteria for AML, and marrow CR is defined as per the IWG criteria for MDS. **Patient 6 only received 2/7 IL2 doses due to the development of hepatotoxicity

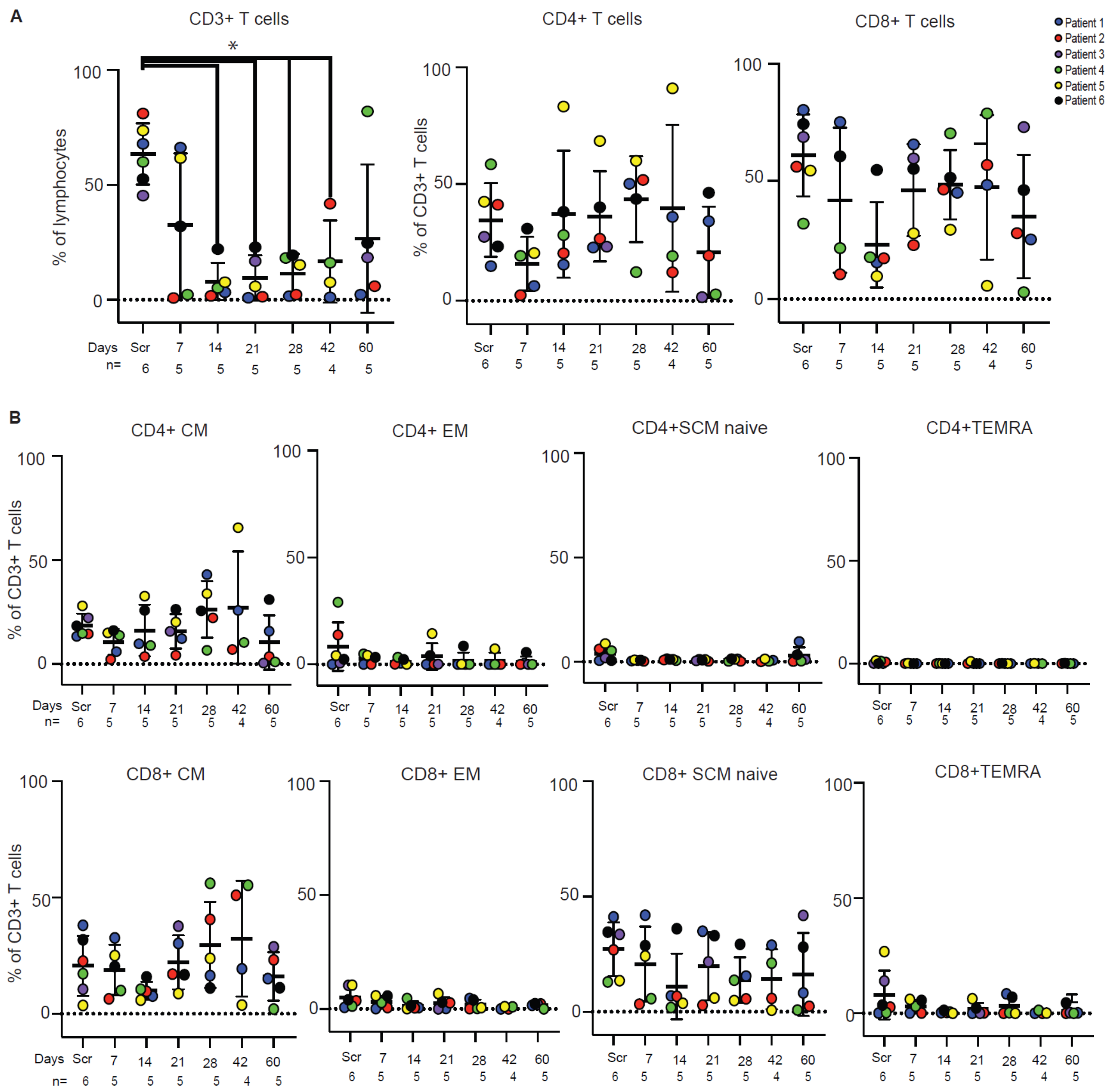

Figure S2. T-cell subpopulations following CIML NK cell infusion. A Longitudinal evaluation of $CD3^+$, $CD4^+$, and $CD8^+$ T-cell subpopulations in all patients using a customized flow cytometry panel. Comparison of abundance of $CD3^+$ T-cell subpopulations at the different time points is shown. *$p<0.05$ Mann-Whitney U test, with significance adjusted by Holm's method for multiple comparisons. **B** Longitudinal evaluation of $CD4^+$ and $CD8^+$ subpopulations, including central memory (CM), effector memory (EM), stem cell memory naive (SCM naive), and effector memory T-cells expressing CD45RA (TEMRA). The abundance of $CD4^+$ and $CD8^+$ T-cell subpopulations at the different time points is shown.

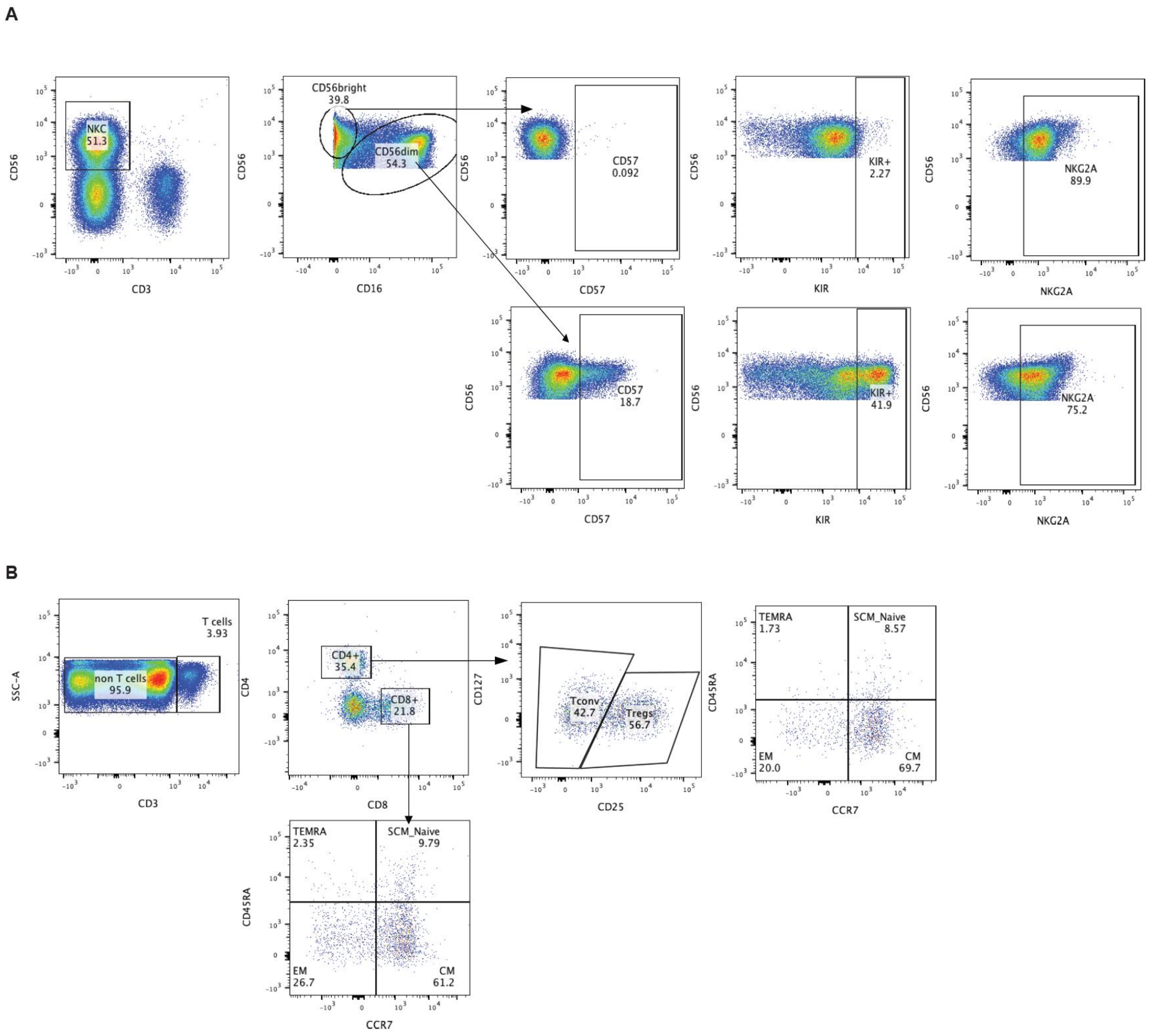

Figure S3. Gating strategies for characterization of NK and T cell subsets by flow cytometry. A NK cells are CD56^{+}CD3^{-}. Bright and dim NK cells are characterized by the expression of CD56 and CD16. The expression of CD57, KIR and NKG2A is determined in bright and dim populations. **B** T cells are CD3^{+}. CD8^{+} and CD4^{+} subsets are characterized by the expression of CD4 and CD8. Conventional CD4^{+} T cells are CD127^{+}CD25^{-}, where CD127^{-}CD25^{+} cells are Tregs. Conventional CD4^{+} and CD8^{+} T cell subsets are characterized by the expression of CD45RA and CCR7 where effector memory (EM) T cells are CD45RA^{-}CCR7^{-}, central memory (CM) T cells are CCR7^{+}CD45RA^{-}, terminally differentiated effector memory T cells (TEMRA) are defined by CD45RA^{+}CCR7^{-} and stem cell memory T cells (SCM_Naive) are characterized as CD45RA^{+}CCR7^{+}. Representative flow plots are patient 5 day +42 and patient 4 day+14, respectively.

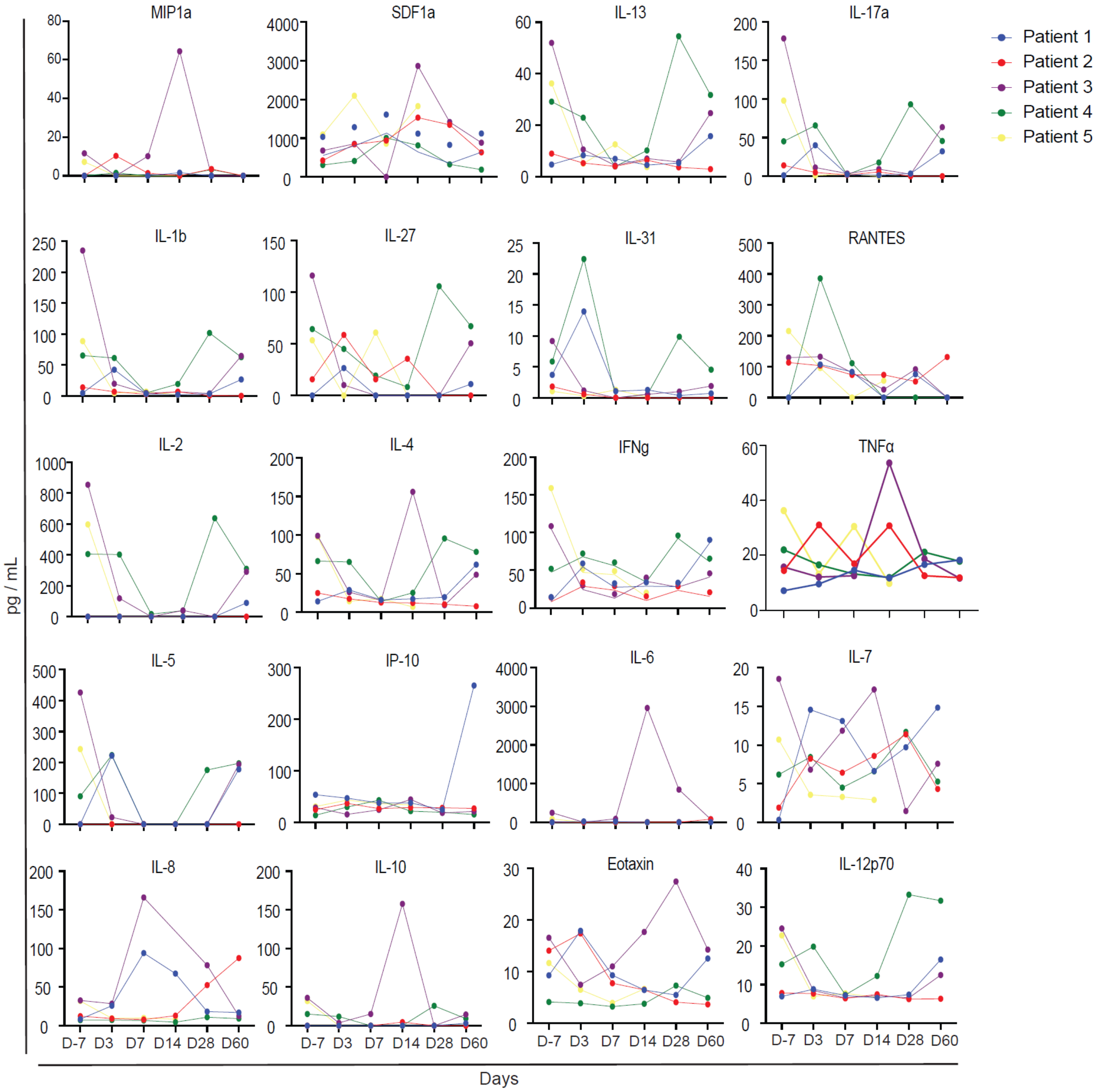

Figure S4. Evaluation of endogenous cytokines in longitudinal serum samples from patients on the CIML NK trial. Each measured serum cytokine is indicated above each graph. Longitudinal time points include day -7 (D-7), day +3 (D3), day +7 (D7), day +14 (D14), day +28 (D28), and day +60 (D60) following CIML NK infusion.

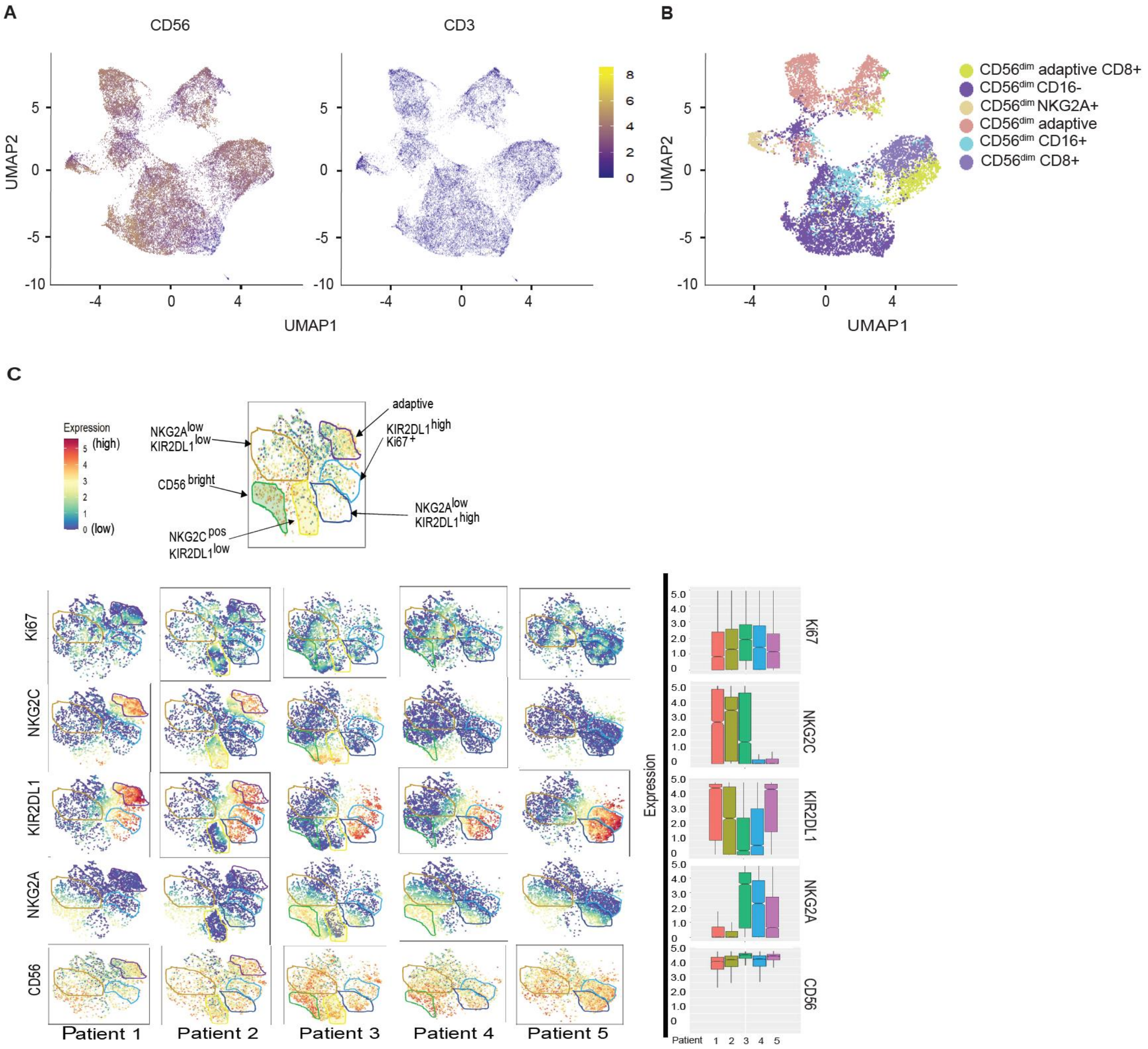

Figure S5. The infusion product contains distinct subpopulations with natural variation in donor NK cell repertoires A The infusion product in all patients is enriched with $CD56^{+}CD3^{-}$ NK cell populations. Shown is the UMAP of immune cell clusters expressing $CD56^{+}$ and $CD3^{-}$ derived from the infusion products of all 6 patients treated with CIML NK cell therapy. **B** Distribution of NK cell clusters among all patient infusion products within the CYTOF marker UMAP space, showing a number of existing CIML NK subpopulations. **C** The NK cell subpopulations in the infusion products are compared against each other in tSNE plots derived from the first 5 patients on the trial, with distinct NK cell subpopulations identified using PhenoGraph clustering. The scale of expression on the tSNE plots is indicated in the top left corner. The scaled expression of the indicated markers is shown as boxplots on the right, with each boxplot corresponding to an individual patient.

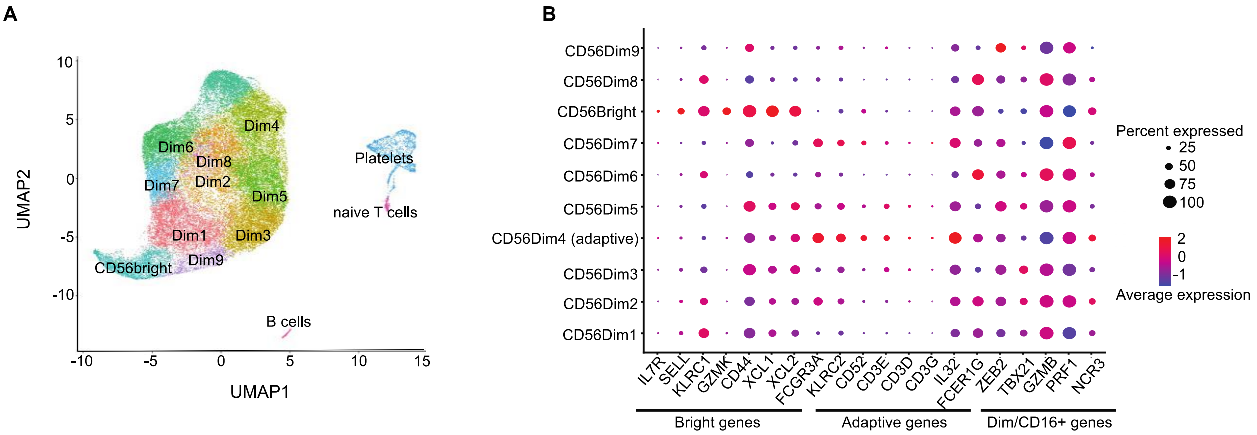

Figure S6. Single cell RNA sequencing characterization of donor infusion products yields distinct NK cell subpopulations A Distribution of NK cell clusters among all patient infusion products within the gene expression UMAP space identifies a number of CD56^{dim} populations among the CIML NK cells. A CD56^{bright} NK cluster is identified that could not be well-characterized with mass cytometry. **B** Dot plot of genes characterizing the individual CD56^{dim} and CD56^{bright} NK subpopulations among all CIML NK infusion products.

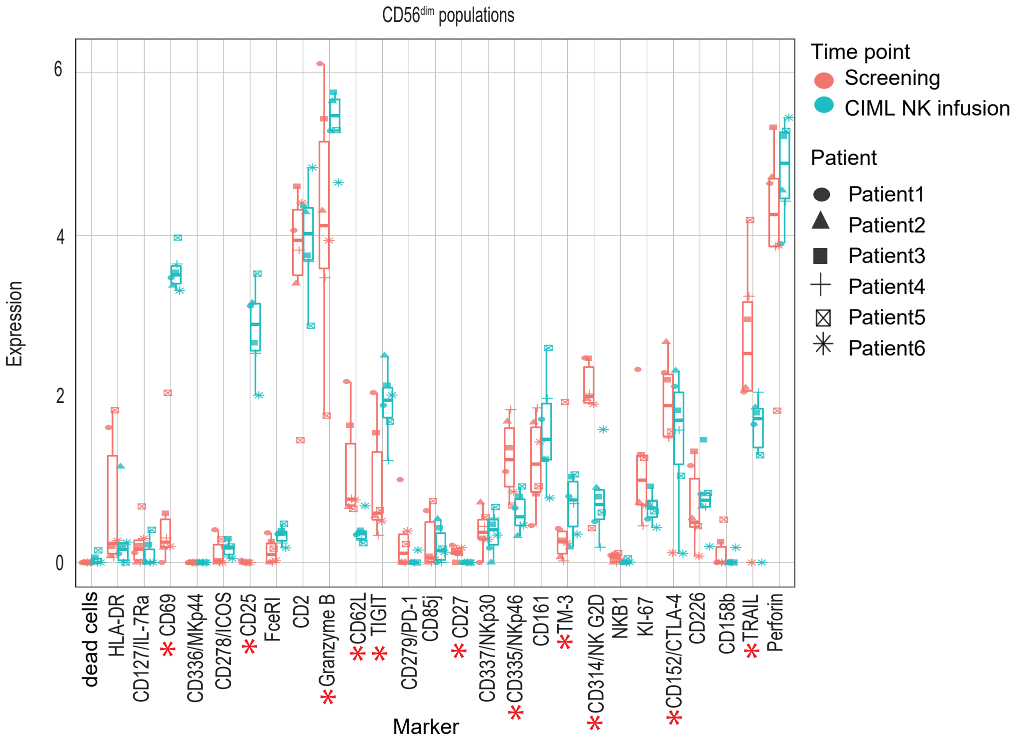

Figure S7. Differential expression analysis of marker expression on CD56^{dim} populations in the infusion product compared to the screening time point. CD56^{dim} NK cells from the screening time point before CIML NK infusion (red) from all patients treated on the trial are compared to the CD56^{dim} NK cells in the infusion product (green). * p<0.05 by Wilcoxon rank sum test, with significance adjusted for multiple comparisons (43).

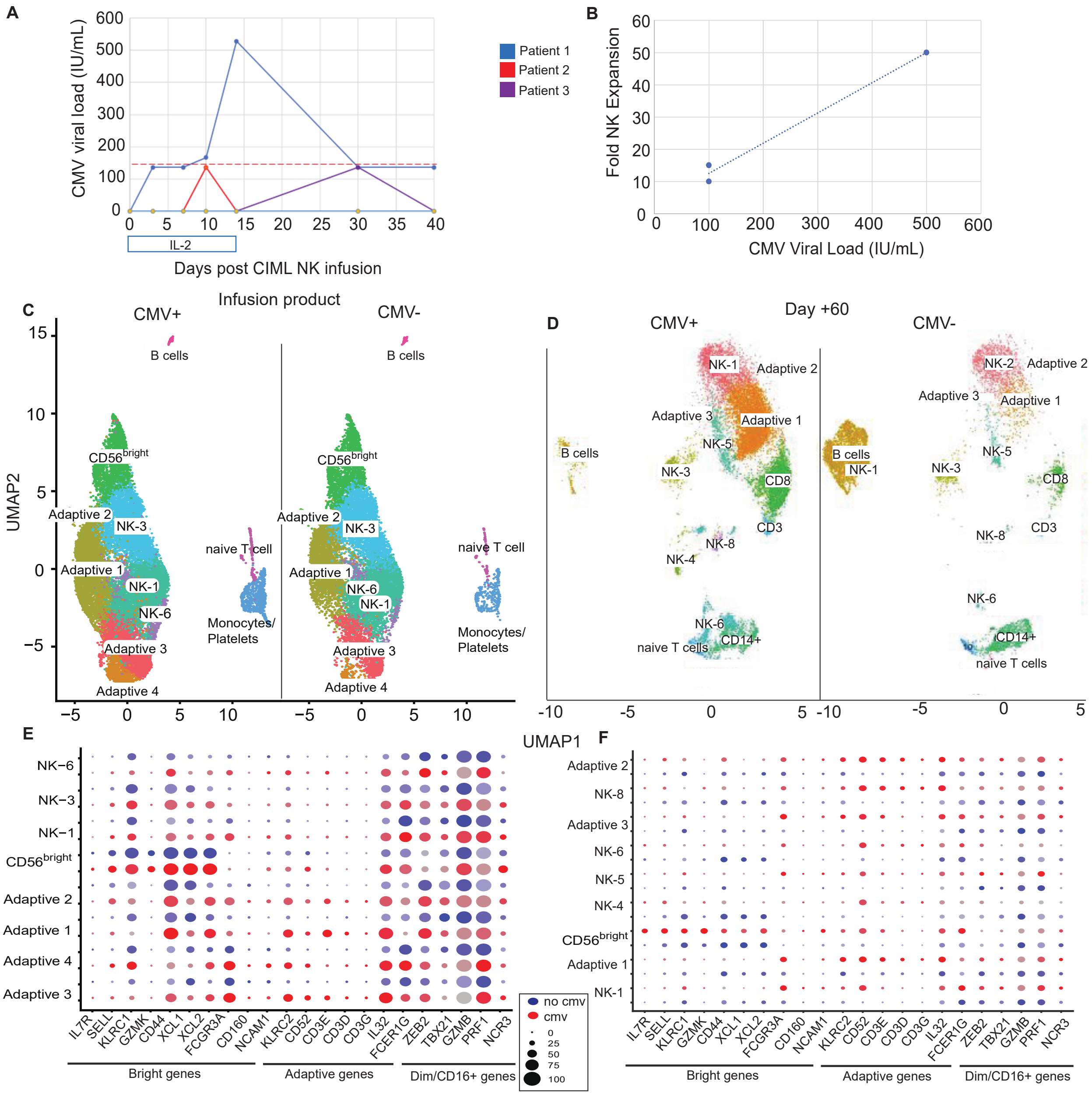

Figure S8. CMV reactivation is associated with expansion of an adaptive NK cell population, while non-adaptive NK cell subpopulations expand independently of CMV. A CMV reactivation occurred in 3/6 treated patients, with viral load measured using a clinical grade assay. **B** Correlation between CMV viral load after reactivation and fold NK expansion in the 3 patients in whom CMV reactivated. **C** Gene expression UMAP of CIML NK infusion products in patients who reactivated CMV (CMV+) compared to those who did not (CMV-), showing a similar distribution of NK cell subpopulations. **D** Gene expression UMAP of NK cell subpopulations at the day +60 time point after CIML NK cell infusion. **E** Dot plot of NK cell subpopulations in the infusion product, showing genes associated with the adaptive NK cell phenotype, the CD56^{bright} NK cell phenotype, and with the CD56^{dim}/CD16^{+} mature NK cell phenotype. Samples from both CMV- (blue dots) and CMV+(red dots) are shown. **F** Dot plot of NK cell subpopulations at day +60 after CIML NK cell infusion, showing persistence of both adaptive and non-adaptive NK cells in both CMV+ and CMV- groups.

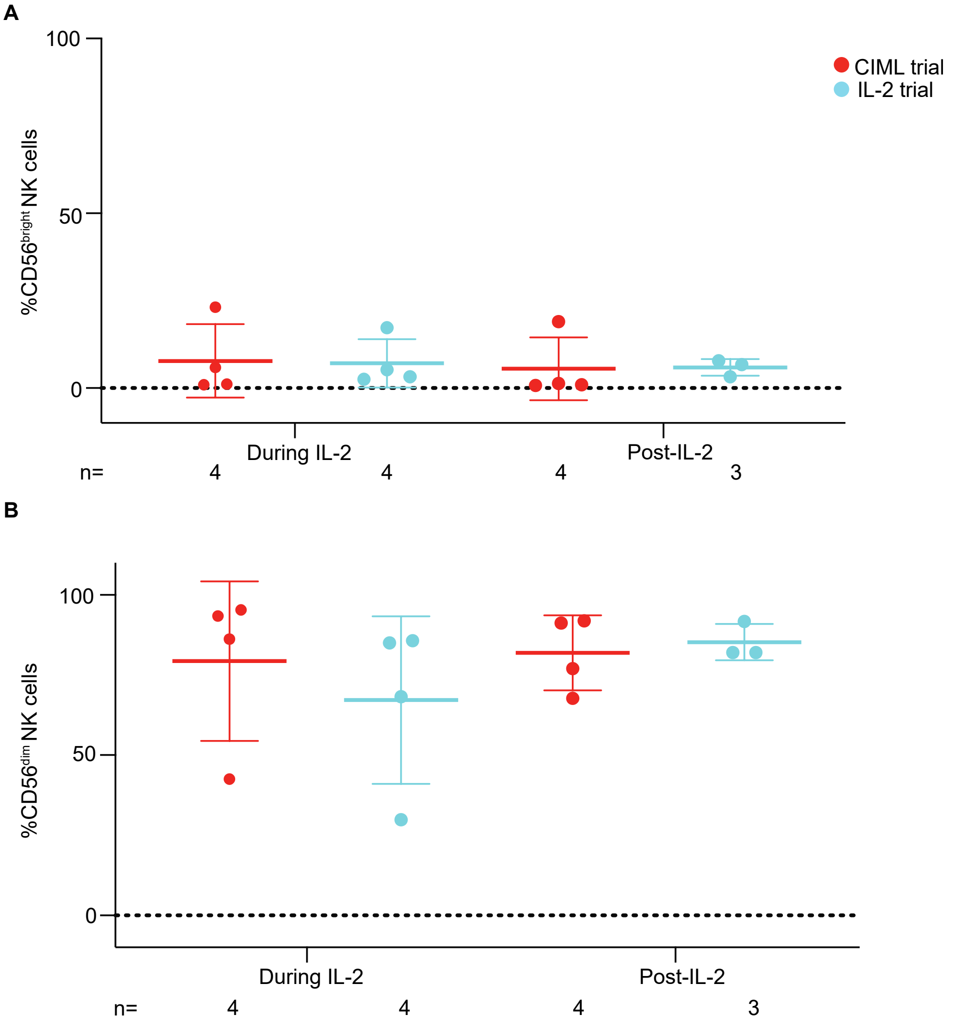

Figure S9. There is no difference in the percentage of peripheral blood NK cells that are CD56^{bright} or CD56^{dim} between post-HCT patients receiving IL-2 and CIML NK infusion patients receiving IL-2. A CD56^{bright} and **B** CD56^{dim} NK cells in the peripheral blood of patients treated with CIML NK cell infusion and post-transplant IL-2 treated patients. Data presented is CD56^{dim} and CD56^{bright} populations as a percentage of total CD56^{+}CD3^{-} lymphocytes as determined by flow cytometry. Mean + SD. $p > 0.05$ by Wilcoxon rank sum test, comparing CIML trial and IL-2 trial samples at each of the indicated time points.

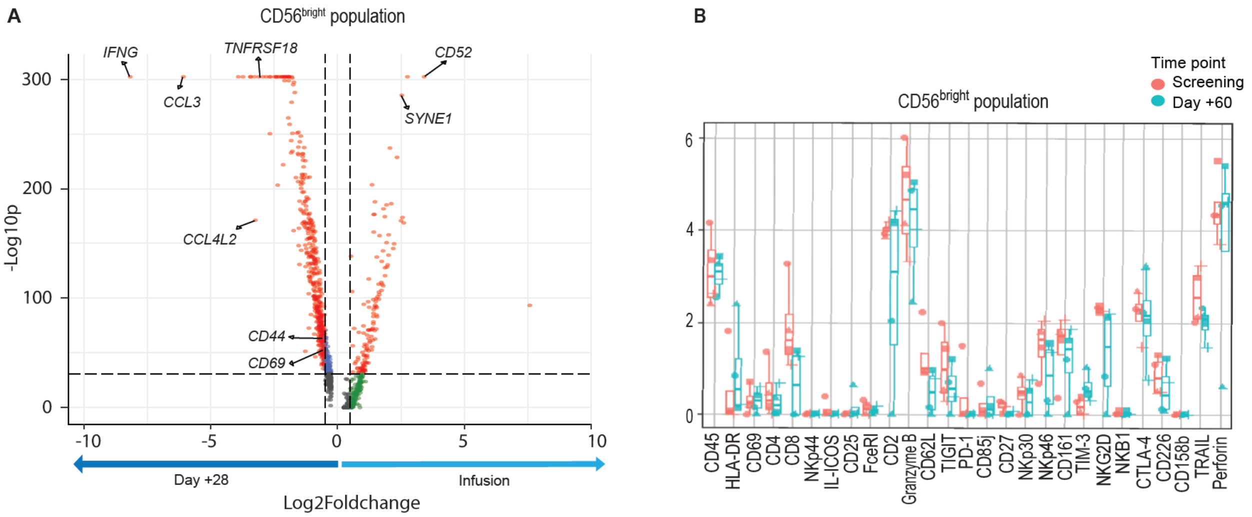

Figure S10. Evaluation of the CD56bright NK cell population following CIML NK infusion. A Volcano plot showing most differentially expressed genes between the CIML NK infusion product to the day+28 time point after infusion of CIML NK cells (p cut off = 10e-32, fold change cut off = 0.5)**.** The Wilcoxon rank sum test was used to determine differentially expressed markers between the two groups, with significance adjusted for multiple comparisons (50). **B** Differential expression analysis of mass cytometry markers comparing the screening time point prior to CIML NK infusion to the day+60 time point after infusion (p > 0.05 by Wilcoxon rank sum test).

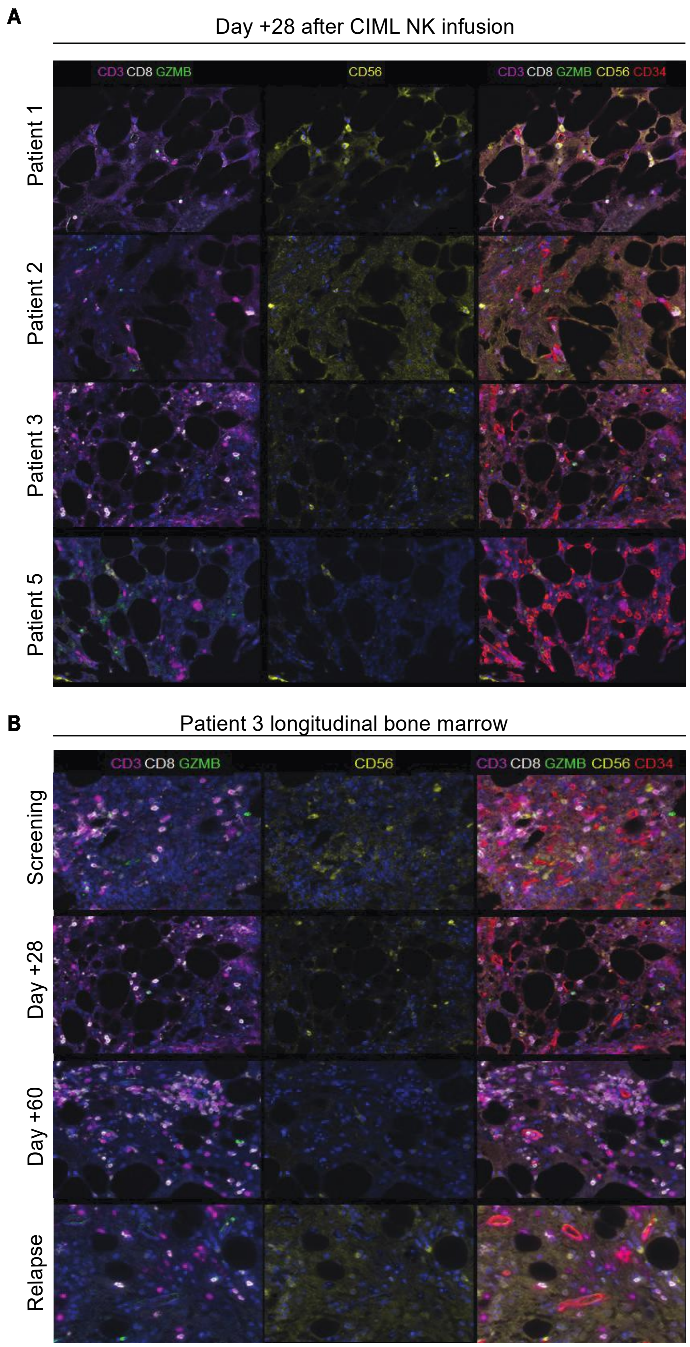

Figure S11. NK cell infiltration into the bone marrow following CIML NK infusion. A Distribution of markers in bone marrow core biopsies from each patient taken at day +28 following CIML NK infusion in patients with bone marrow disease. Shown are $CD3^+$ cells (purple), $CD8^+$ cells (white), $CD56^+$ cells (yellow), and juxtaposition of these cells next to $CD34^+$ cells (blasts). **B** Longitudinal bone marrow core samples in representative patient 3. The time points correspond to screening (prior to CIML NK infusion), day +28 following CIML NK, day +60 following CIML NK infusion, and relapse (day +120 following CIML NK infusion).

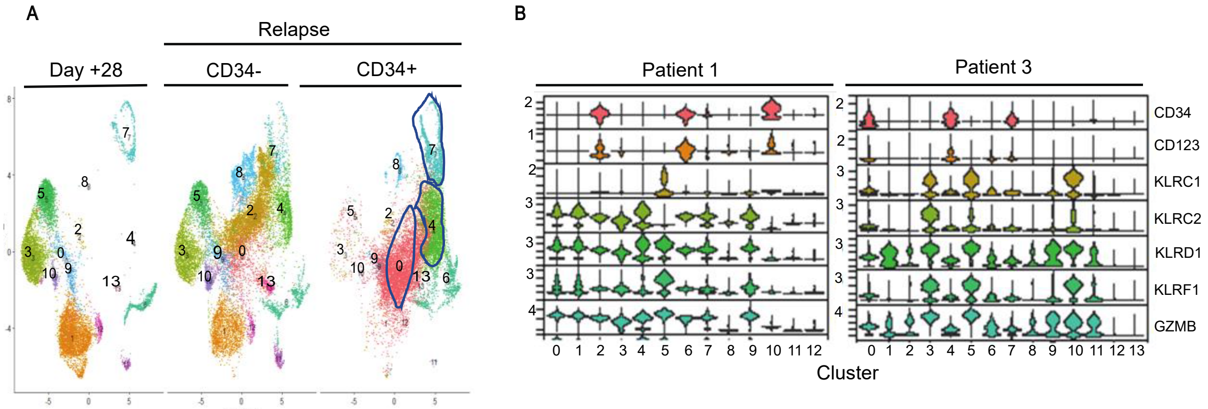

Figure S12. Identification of CD34$^+$ blasts relapsed following CIML NK therapy in patient. A Representative UMAP plot of scRNAseq data applied to both CD34$^-$ and CD34$^+$ fractions of post-CIML NK relapse sample from patient 3. The clusters at relapsed are compared to the clusters at day +28 following CIML NK cell infusion. Clusters 0, 4, and 7 represent relapsed leukemia blasts. **B** Violin plots corresponding to relapse clusters in both patient 1 and patient 3, both of whom had CD34$^+$CD123$^+$ blasts in the peripheral blood. The remaining clusters express NK cell genes.

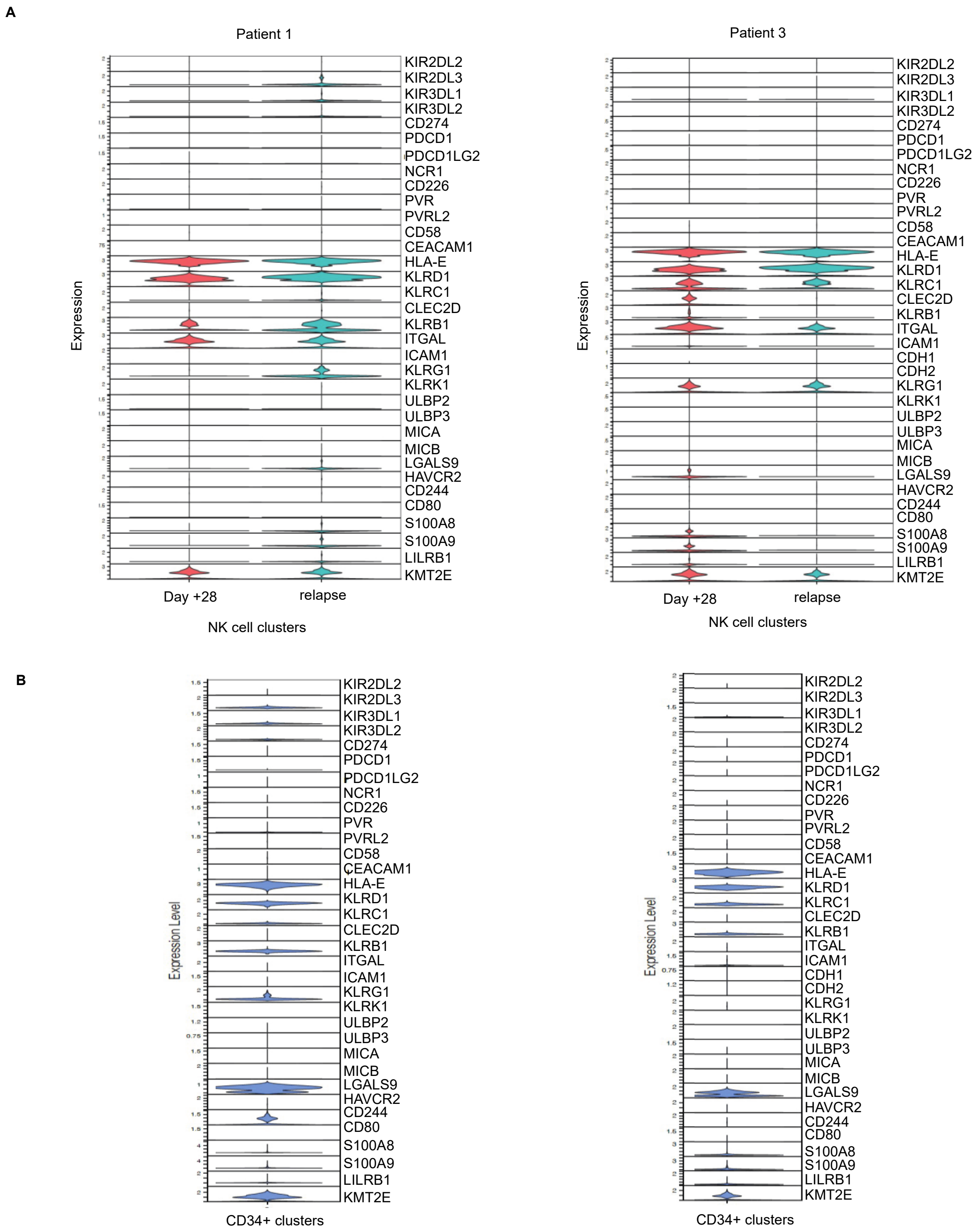

Figure S13. Single-cell RNA sequencing of peripheral blood mononuclear cells from Patient 1 (left) and Patient 3 (right) at the day +28 and relapse time points, with the cells from the latter fraction separated into CD34-depleted (CD34^{-}) fractions (A) and CD34-enriched (CD34^{+}) fractions (B). A Within the CD34-depleted fraction, NK cell clusters were identified based on the expression of the markers *NCAM1, KLRD1, KLRF1, NCR1, NKG7, GNLY, CD3D, CD3E*, and *CD3G*. Violin plots show gene expression of known NK receptors on the NK cell clusters in both patients. **B** Within the CD34-enriched fraction, clusters corresponding to leukemia blasts were identified based on the expression of CD34 and CD123, and absence of the aforementioned NK cell markers. Violin plots show the expression of known NK activating and inhibitory ligands on leukemia blasts in Patient 1 (left) and Patient 3 (right).

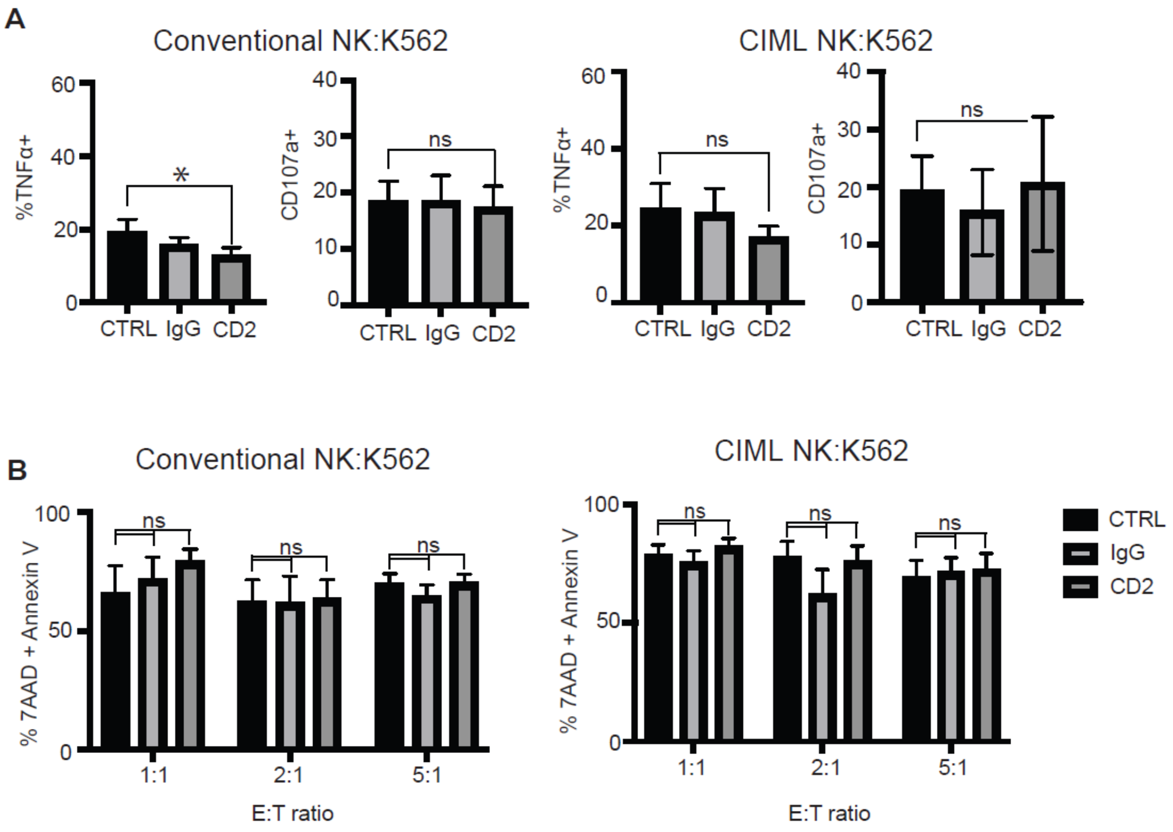

Figure S14. CD2 blockade is not sufficient to alter the function of CIML NK cells in vitro. A Conventional and CIML NK cells from healthy donors were preincubated with an anti-human mAb against CD2 or an IgG1 isotype control for 30 minutes before co-culture with K562 for 6 hours at an E:T ratio of 5:1. Data presented show the mean percentage ± SEM of NK cells that were positive for TNFα and CD107a. n= 6 healthy donors in 2 independent experiments. **B** Following preincubation for 30 minutes with mAb against CD2 or an IgG1 isotype control, CIML and conventional NK cells were co-cultured with K562 at 3 E:T ratios for 4 hours. Data presented is the mean percentage ± SEM of 7AAD and Annexin V positive K562 target cells. N=6 healthy donors in 2 independent experiments. * $p<0.05$ with Wilcoxon matched pairs signed rank test

Table S1. Clinical characteristics and outcomes of patients treated with CIML NK cells

Patient	Diagnosis[A]	Treatment prior to CIML NK (#months relative to HCT)[B]	CMV serostatus and reactivation[C]	Response at Day +28[D]	Adverse Events
Patient 1	Secondary AML with FLT3-ITD	(-3m) 3+7 + Midostaurin<br>(0m) RIC PBSC HCT<br>(8m) Relapse<br>(9m) Gilteritinib<br>(11m) added Decitabine<br>(12m) added Venetoclax<br>(14m) CIML NK	Donor: **seropositive**<br>Recipient: **seronegative**<br>Day of reactivation post-CIML NK: **day +4[D]**	Morphologic leukemia-free state	Fever<br>Prolonged pancytopenia requiring stem cell boost
Patient 2	MDS with excess blasts II, multiple pathogenic mutations	(-8m) AZA x 2<br>(-5m) AZA + Venetoclax x 2<br>(0m) RIC PBSC HCT<br>(7m) Relapse<br>(8m) CIML NK	Donor: **seropositive**<br>Recipient: **seropositive**<br>Day of reactivation post-CIML NK: **day +10**	Complete Remission	Fever<br>Prolonged pancytopenia requiring stem cell boost<br>Fungal infection
Patient 3	Secondary AML with TP53 mutation, multiple other pathogenic mutations	(-4m) 3+7<br>(0m) RIC PBSC HCT<br>(14m) Relapse<br>(15m) CIML NK	Donor: **seropositive**<br>Recipient: **seropositive**<br>Day of reactivation post-CIML NK: **day +20**	Complete Remission	Cytokine Release Syndrome<br>Pancytopenia
Patient 4	BPDCN	(-2m) Tagraxofusp<br>(0m) RIC PBSC HCT<br>(10m) Relapse<br>(11m) CIML NK	Donor: **seronegative**<br>Recipient: **seronegative**<br>Day of reactivation post-CIML NK: **no reactivation**	Complete Remission	Fever
Patient 5	Secondary AML with multiple pathogenic mutations	(-34m) clinical trial #1[E]<br>(-28m) RIC DUCBT<br>(-11m) Relapse<br>(-11m) clinical trial #2<br>(-9m) clinical trial #3<br>(-6m) Decitabine + Venetoclax	Donor: **seronegative**<br>Recipient: **seronegative**<br>Day of reactivation post-CIML NK: **no reactivation**	Refractory to therapy	Fever

		(0m) RIC PBSC HCT<br>(18m) Relapse<br>(20m) CIML NK			
Patient 6	CML with blast crisis	(-30m) Dasatinib<br>(-6m) 3+7 + imatinib<br>(0m) MAC PBSC HCT<br>(4m) Relapse<br>(5m) Ponatinib<br>(7m) CIML NK	Donor: **seropositive**<br>Recipient: **seropositive**<br>Day of reactivation post-CIML NK: **no reactivation**	Refractory to therapy	Elevations in ALT and AST during IL-2 injections

[A] AML: acute myeloid leukemia, MDS: myelodysplastic syndrome, BPDCN: blastic plasmacytic dendritic cell neoplasm, 3+7: induction chemotherapy with daunorubicin and cytarabine, AZA: azacytidine, RIC: reduced intensity conditioning, PBSC: peripheral blood stem cell graft, DUCBT: double-umbilical cord blood transplant
[B] Refers to sequence of therapy and disease status prior to CIML NK cell therapy. The date of treatment or disease status is indicated as relative to the date of haploidentical stem cell transplant (HCT), and is reported in the number of months. Dates prior to HCT are reported with a negative number of months.
[C] Refers to CMV reactivation as measured with a clinical grade viral load PCR assay, with the time indicated relative to Day 0, the day of CIML NK cell infusion
[D] Refers to number of days from the date of CIML NK infusion, which is day 0
[E] The therapy administered in all prior clinical trials did not meet any exclusion criteria for the CIML NK phase I trial

Table S2: Top 50 differentially expressed genes in CD56^{dim} NK cell clusters between infusion and day + 28

	p_val	avg_log2FC	pct.1	pct.2	p_val_adj
**SH3BGRL3**	0	1.65331181	0.974	0.888	0
**CD52**	0	3.97972095	0.948	0.172	0
**TXNIP**	0	3.0052993	0.983	0.623	0
**S100A6**	0	1.66046365	0.884	0.613	0
**S100A4**	0	2.61905797	0.953	0.415	0
**ZFP36L2**	0	1.83817706	0.772	0.387	0
**CXCR4**	0	1.55517544	0.44	0.075	0
**SPON2**	0	1.92245792	0.486	0.066	0
**FGFBP2**	0	2.44657005	0.797	0.31	0
**PLAC8**	0	1.9099973	0.688	0.205	0
**GZMK**	0	1.89216035	0.282	0.082	0
**GZMA**	0	2.11772155	0.934	0.596	0
**HLA-DRB1**	0	1.74417085	0.702	0.276	0
**HLA-DPA1**	0	1.55767892	0.716	0.383	0
**HLA-DPB1**	0	1.83412091	0.75	0.264	0
**SYNE1**	0	1.80584775	0.611	0.106	0
**ANXA1**	0	1.93570063	0.836	0.322	0
**CLIC3**	0	2.27539824	0.664	0.113	0
**IFITM2**	0	2.37491916	0.946	0.511	0
**LSP1**	0	1.59845272	0.732	0.274	0
**HBB**	0	2.01463116	0.268	0.006	0
**AHNAK**	0	1.90097637	0.722	0.19	0
**CTSW**	0	1.59840065	0.918	0.618	0
**PTPRCAP**	0	2.49806396	0.511	0.001	0
**CD3E**	0	1.78442371	0.614	0.177	0
**KLRF1**	0	1.54904867	0.566	0.218	0
**C12orf75**	0	1.54173437	0.693	0.27	0
**CYBA**	0	1.64655632	0.987	0.832	0
**CCL5**	0	1.91953293	0.934	0.769	0
**GZMM**	0	1.82067048	0.731	0.293	0
**MYO1F**	0	1.54492026	0.611	0.128	0
**KLF2**	0	1.81982876	0.876	0.401	0
**HCST**	0	2.16144266	0.947	0.699	0
**TYROBP**	0	2.26032535	0.899	0.531	0
**NKG7**	0	1.71676172	0.98	0.975	0
**ITGB2**	0	1.54818858	0.814	0.432	0
**C1orf63**	0	1.68819122	0.382	0	0
**ATPIF1**	0	1.77427207	0.42	0	0
**ATP5I**	0	1.58326068	0.375	0	0
**GNB2L1**	0	3.06501041	0.556	0	0
**SEPT7**	0	2.24526951	0.487	0	0
**RARRES3**	0	2.15968985	0.466	0	0
**ATP5L**	0	2.39470426	0.508	0	0
**ATP5G2**	0	1.88736811	0.425	0	0
**TCEB2**	0	2.1870572	0.492	0	0
**ATP5E**	0	3.55722961	0.573	0	0
**ATP5D**	0	1.54817761	0.372	0	0
**UQCR11.1**	0	1.98973427	0.452	0	0
**AES**	0	2.44035938	0.514	0	0
**GLTSCR2**	0	1.55772267	0.363	0	0

Table S3: Top 50 differentially expressed genes in $CD56^{bright}$ NK cell clusters between infusion and day +28

	p_val	avg_log2FC	pct.1	pct.2	p_val_adj
**CD52**	0	3.41543025	0.716	0.064	0
**TXNIP**	0	2.75710844	0.893	0.432	0
**SYNE1**	1.46E-290	2.53441101	0.703	0.142	4.07E-286
**AHNAK**	2.43E-242	2.06382346	0.721	0.238	6.80E-238
**FGFBP2**	7.89E-234	2.34423541	0.66	0.175	2.21E-229
**FLNA**	3.55E-193	1.47454092	0.817	0.546	9.92E-189
**CD3E**	1.64E-191	2.11837858	0.55	0.11	4.58E-187
**MYO1F**	2.23E-190	1.93276621	0.598	0.154	6.23E-186
**ZFP36L2**	9.08E-187	1.81076321	0.719	0.369	2.54E-182
**SYNE2**	1.05E-181	1.4538044	0.807	0.512	2.93E-177
**LSP1**	1.22E-179	1.86455679	0.617	0.214	3.43E-175
**GNB2L1**	6.42E-179	2.56115414	0.412	0	1.80E-174
**AES**	7.02E-176	2.50267211	0.406	0	1.97E-171
**ATP5E**	6.33E-174	2.60946411	0.403	0	1.77E-169
**PPP2R5C**	1.48E-165	1.42645973	0.764	0.478	4.14E-161
**CEP78**	5.14E-163	2.01447744	0.532	0.138	1.44E-158
**ITGAL**	2.92E-162	1.65468092	0.646	0.277	8.18E-158
**S100A4**	6.60E-156	1.93165464	0.617	0.249	1.85E-151
**CXCR4**	8.46E-155	2.2292528	0.428	0.046	2.37E-150
**C1orf63**	1.64E-150	2.19578669	0.359	0	4.60E-146
**PIK3R1**	2.20E-148	1.79271327	0.631	0.312	6.17E-144
**SPON2**	1.73E-144	2.01129084	0.416	0.05	4.85E-140
**EVL**	1.01E-142	1.54721041	0.633	0.294	2.82E-138
**GZMM**	9.99E-139	1.74313164	0.529	0.186	2.80E-134
**SEPT7**	1.36E-135	1.97662615	0.33	0	3.81E-131
**PTPRCAP**	1.14E-133	1.9471082	0.326	0	3.19E-129
**CLIC3**	6.07E-133	1.87405125	0.418	0.068	1.70E-128
**RNF166**	1.18E-125	1.87182167	0.4	0.066	3.31E-121
**IFITM2**	1.55E-119	1.7243972	0.534	0.233	4.35E-115
**SIGIRR**	5.46E-118	1.75242777	0.396	0.079	1.53E-113
**ADD3**	7.17E-118	1.6197146	0.472	0.144	2.01E-113
**TMEM2**	1.72E-116	1.79550939	0.291	0	4.81E-112
**MIR142**	3.02E-112	1.73403551	0.282	0	8.44E-108
**MATR3**	1.09E-110	1.63500419	0.28	0.001	3.04E-106
**GLTSCR2**	1.68E-108	1.64528312	0.274	0	4.71E-104
**ATP5L**	2.02E-106	1.58086705	0.269	0	5.66E-102
**TRAF3IP3**	1.01E-104	1.52752185	0.408	0.109	2.83E-100
**FYB**	3.32E-104	1.62696743	0.265	0	9.29E-100
**TCEB2**	5.27E-102	1.50250187	0.26	0	1.48E-97
**RARRES3**	1.17E-98	1.48095953	0.252	0	3.28E-94
**HBB**	1.50E-98	7.57984421	0.259	0.004	4.20E-94
**ANXA1**	1.61E-97	1.52293179	0.452	0.169	4.52E-93
**TSPAN32**	7.88E-97	1.46555292	0.26	0.007	2.21E-92
**RASGRP2**	1.67E-96	1.54118622	0.281	0.02	4.67E-92
**SAMD3**	7.50E-95	1.42581733	0.419	0.136	2.10E-90
**RNF125**	8.22E-90	1.53670272	0.345	0.078	2.30E-85
**RBL2**	3.85E-88	1.55776093	0.368	0.106	1.08E-83
**CNOT6L**	5.38E-77	1.52702371	0.431	0.194	1.51E-72
**ATG2A**	1.76E-75	1.46246015	0.292	0.061	4.92E-71
**GZMA**	7.85E-6	1.47955672	0.556	0.359	2.20E-63

Table S4: Top 50 differentially expressed genes in adaptive NK cell clusters between cmv+ and cmv- groups

	p_val	avg_log2FC	pct.1	pct.2	p_val_adj
**AC092580.4**	0	1.4779754	0.359	0	0
**AES**	0	2.76725441	0.648	0	0
**ATP5D**	0	1.7596341	0.452	0	0
**ATP5E**	0	3.83398683	0.717	0	0
**ATP5G2**	0	2.12903297	0.522	0	0
**ATP5G3**	0	1.62384338	0.414	0	0
**ATP5I**	0	1.78379868	0.453	0	0
**ATP5J**	0	1.53747166	0.398	0	0
**ATP5J2**	0	1.63283646	0.425	0	0
**ATP5L**	0	2.64266367	0.621	0	0
**ATPIF1**	0	1.97915341	0.496	0	0
**C14orf2**	0	1.63054298	0.421	0	0
**C19orf43**	0	1.76281172	0.448	0	0
**C1orf63**	0	2.0174922	0.487	0	0
**C9orf142**	0	1.64491835	0.413	0	0
**CD3E**	0	1.29820465	0.757	0.325	0
**FAM65B**	0	1.49932932	0.364	0	0
**FYB**	0	1.65372855	0.397	0	0
**GLTSCR2**	0	1.82301591	0.46	0	0
**GNB2L1**	0	3.33028553	0.7	0	0
**GPR56**	0	1.44789096	0.341	0	0
**IL32**	0	1.32768016	0.954	0.562	0
**KIAA1551**	0	1.42232532	0.339	0	0
**KLRC2**	0	1.62574862	0.663	0.242	0
**MATR3**	0	1.66987422	0.414	0	0
**MINOS1**	0	1.35789976	0.35	0	0
**MIR142**	0	1.69421205	0.403	0	0
**MYEOV2**	0	1.5530903	0.409	0	0
**NDUFB8.1**	0	1.66278626	0.434	0	0
**PTPRCAP**	0	2.75664284	0.634	0.001	0
**RARRES3**	0	2.36319521	0.569	0	0
**SEPT7**	0	2.48928405	0.6	0	0
**SEPW1**	0	1.38196054	0.353	0	0
**SF3B14**	0	1.26828252	0.332	0	0
**TCEB2**	0	2.43125446	0.6	0	0
**TMEM2**	0	1.53594732	0.355	0	0
**TMEM66**	0	1.58271183	0.401	0	0
**UQCR11.1**	0	2.19370078	0.55	0	0
**USMG5**	0	1.66462395	0.431	0	0
**TMBIM4.1**	2.070E-321	1.21335159	0.31	0	5.42382E-317
**SELK**	2.500E-321	1.22242695	0.31	0	6.5527010E-317
**WHSC1L1**	9.097E-320	1.25582106	0.308	0	2.38920571E-315
**LSMD1**	1.8320168783E-313	1.18548997	0.303	0	0.00E+00
**ALDOA**	0.00E+00	1.35181772	0.441	0.126	1.02E-304
**U2AF1**	4.19E-308	1.33559683	0.381	0.039	1.10E-303
**NHP2L1**	2.78E-307	1.16647545	0.298	0	7.29E-303
**C20orf24**	1.05E-301	1.19194305	0.294	0	2.75E-297
**MGEA5**	2.45E-300	1.24916678	0.292	0	6.43E-296
**SEPT9**	9.86E-291	1.19018818	0.284	0	2.59E-286
**CD3D**	7.70E-183	1.45615361	0.315	0.053	2.02E-178

Table S5: Top 50 differentially expressed genes in NK cell clusters between day + 28 and relapse Patient 1

	p_val	avg_log2FC	pct.1	pct.2	p_val_adj
**HBA1**	0	0.6608472	0.686	0.041	0
**HBA2**	0	0.73567322	0.682	0.007	0
**HBB**	0	0.68960964	0.644	0.001	0
**HBG1**	0	0.45516876	0.331	0	0
**HBG2**	0	0.69251925	0.417	0	0
**FOS**	4.28E-229	0.30346976	0.614	0.289	8.55E-226
**SNORD3B-2**	2.23E-115	0.32173532	0.333	0.116	4.46E-112
**HBA11***	0	0.64873468	0.707	0.038	0
**HBA21**	0	0.58470732	0.674	0.007	0
**HBB1**	0	0.4813301	0.677	0.002	0
**HBG21**	2.9881E-319	0.76336277	0.386	0	5.97621810E-316
**HBG11**	4.59E-251	0.61967926	0.312	0	9.19E-248
**AKR1C3**	1.84E-211	0.31075672	0.479	0.132	3.69E-208
**SNORD3A1**	3.86E-97	0.38469356	0.39	0.148	7.72E-94
**WDR74**	2.58E-91	0.33721122	0.497	0.26	5.17E-88
**SNORD3B-21**	7.40E-63	0.3035759	0.227	0.066	1.48E-59
**HBB2**	5.02E-215	0.63773936	0.785	0.005	1.00E-211
**HBA22**	4.52E-192	0.83533409	0.73	0.004	9.05E-189
**YWHAH**	1.68E-183	0.32109328	0.752	0.056	3.36E-180
**HBA12**	3.78E-170	0.63136327	0.693	0.02	7.56E-167
**LGALS3**	4.31E-165	0.31754888	0.804	0.141	8.63E-162
**IGFBP7**	7.42E-165	0.38121629	0.739	0.074	1.48E-161
**RANBP1**	2.31E-160	0.30437346	0.718	0.073	4.63E-157
**C1orf561**	2.14E-137	0.31171922	0.856	0.265	4.29E-134
**CCL3**	2.80E-104	0.41372658	0.549	0.063	5.60E-101
**RNU12**	5.93E-102	0.36551292	0.46	0.011	1.19E-98
**HBG22**	2.90E-89	1.16468274	0.38	0	5.80E-86
**FOSB**	5.94E-86	0.59769954	0.567	0.101	1.19E-82
**TNFRSF18**	3.10E-76	0.30856559	0.669	0.247	6.19E-73
**HIST1H2AC**	3.06E-72	0.32513061	0.417	0.048	6.11E-69
**HBG12**	1.42E-70	0.51878803	0.307	0	2.83E-67
**FOS1**	4.17E-67	0.4331315	0.497	0.117	8.33E-64
**IER2**	3.53E-63	0.33575119	0.712	0.308	7.06E-60
**SLAMF7**	8.63E-48	0.34283179	0.448	0.133	1.73E-44
**TRIP11**	8.26E-44	0.31395986	0.371	0.092	1.65E-40
**PYCARD**	7.87E-29	0.57527888	0.31	0.069	1.57E-25
**SNORD3B-22**	1.48E-26	0.41087487	0.261	0.055	2.95E-23
**CCL4**	2.71E-25	0.84084499	0.574	0.287	5.41E-22
**HSPA1A**	2.02E-23	0.52299782	0.175	0.028	4.04E-20
**HBB3**	2.24E-140	0.59562776	0.687	0.002	4.48E-137
**HBA23**	3.31E-131	0.42161268	0.649	0.006	6.62E-128
**HBA13**	7.55E-110	0.31291468	0.632	0.037	1.51E-106
**IFNG**	5.32E-103	0.39581654	0.675	0.085	1.06E-99
**PTGDS**	1.62E-93	0.6093024	0.57	0.051	3.23E-90
**HBG23**	1.12E-78	0.61712815	0.427	0	2.24E-75
**FCRL3**	6.74E-70	0.32678229	0.512	0.064	1.35E-66
**PPP1R12A**	1.59E-66	0.31396117	0.874	0.423	3.18E-63
**HBG13**	1.62E-66	0.56972567	0.368	0	3.24E-63
**SNORD3B-23**	2.60E-26	0.50264403	0.427	0.131	5.21E-23
**PPP1R101**	6.38E-25	0.35182414	0.594	0.3	1.28E-21

* Genes that are differentially expressed in more than one NK cell cluster have a numerical digit appended at the end of their name corresponding to the order of their appearance

Table S6: Top 50 differentially expressed genes in NK cell clusters between day + 28 and relapse patient 3

	p_val	avg_log2FC	pct.1	pct.2	p_val_adj
**RPS18**	0	0.700509	1	1	0
**RPS6**	0	0.76173785	0.999	0.997	0
**EEF1B2**	2.65E-210	0.77566438	0.982	0.943	5.06E-206
**GIMAP7**	5.59E-199	1.11587312	0.76	0.466	1.07E-194
**TXNIP**	3.77E-184	0.82871694	0.964	0.898	7.20E-180
**PIM1**	1.06E-70	0.70949004	0.31	0.115	2.02E-66
**CD63**	1.17E-67	0.67785939	0.614	0.434	2.24E-63
**JUN**	6.02E-61	0.86009722	0.635	0.461	1.15E-56
**IL7R**	5.40E-54	0.87082682	0.437	0.322	1.03E-49
**FOS**	4.56E-44	0.73085073	0.52	0.357	8.70E-40
**GNLY**	1.79E-30	0.93158689	0.474	0.339	3.43E-26
**KLRD11***	7.49E-121	0.66830854	0.923	0.827	1.43E-116
**GIMAP71**	8.22E-70	0.67472664	0.696	0.484	1.57E-65
**CCL4**	1.11E-66	0.80084414	0.815	0.673	2.13E-62
**JUNB**	9.06E-65	0.74750387	0.644	0.476	1.73E-60
**TSC22D31**	3.29E-60	0.69771497	0.582	0.39	6.29E-56
**FOS1**	6.89E-53	0.79958494	0.454	0.254	1.32E-48
**JUN1**	2.77E-48	0.80594125	0.509	0.33	5.28E-44
**GIMAP72**	3.12E-57	0.79535422	0.661	0.406	5.96E-53
**CCL41**	6.91E-52	1.00953839	0.884	0.772	1.32E-47
**GIMAP12**	2.33E-43	0.71288445	0.294	0.1	4.45E-39
**CCL31**	8.79E-33	1.00561965	0.381	0.223	1.68E-28
**JUN2**	6.01E-29	0.75015941	0.527	0.341	1.15E-24
**FOS2**	1.10E-24	0.66940254	0.537	0.375	2.10E-20
**PTGDS**	1.59E-14	1.19419469	0.158	0.093	3.03E-10
**RPS183**	6.93E-125	0.75125554	1	1	1.32E-120
**RPL133**	4.01E-119	0.71053855	1	1	7.66E-115
**TPT13**	3.73E-103	0.85552981	1	1	7.12E-99
**RPS63**	3.19E-91	0.75093638	1	1	6.08E-87
**EEF1B23**	3.55E-84	0.93643545	0.991	0.983	6.78E-80
**RPL83**	2.24E-80	0.751967	0.999	0.994	4.28E-76
**IL7R1**	1.04E-74	2.15841253	0.735	0.251	1.99E-70
**RPS53**	3.00E-60	0.68805089	0.996	0.992	5.73E-56
**XCL1**	1.85E-46	1.42752161	0.613	0.179	3.53E-42
**XCL22**	3.45E-46	1.36426031	0.797	0.452	6.58E-42
**FOS3**	1.00E-43	1.41131223	0.763	0.581	1.91E-39
**KLRD13**	3.83E-41	0.77951748	0.938	0.915	7.31E-37
**GIMAP73**	1.07E-39	0.86547934	0.72	0.642	2.04E-35
**TSC22D33**	3.80E-28	0.87787925	0.745	0.609	7.26E-24
**CMC11**	1.02E-27	0.87037086	0.854	0.705	1.95E-23
**SPTSSB**	2.44E-27	0.98625473	0.32	0.047	4.67E-23
**HIST1H4C3**	2.36E-25	0.69646004	0.642	0.545	4.51E-21
**JUN3**	4.95E-24	1.00508101	0.695	0.559	9.44E-20
**DUSP13**	1.07E-23	0.85891481	0.588	0.479	2.04E-19
**TNFRSF18**	1.74E-23	0.91695844	0.445	0.163	3.32E-19
**MAL**	5.15E-23	0.9940858	0.231	0.025	9.82E-19
**LTB2**	2.86E-22	1.18456058	0.604	0.366	5.46E-18
**XBP13**	2.91E-19	0.69847236	0.636	0.488	5.56E-15
**C1orf1623**	1.70E-16	0.71063648	0.527	0.298	3.24E-12
**EVA1B**	3.57E-14	0.78676492	0.299	0.14	6.82E-10

* Genes that are differentially expressed in more than one NK cell cluster have a numerical digit appended at the end of their name corresponding to the order of their appearance

Table S7: Flow cytometry antibodies for NK cell subsets

Antibody	Fluorochrome	Clone	Amount (uL)	Vendor	Catalog #
CD16	FITC	3G8	2.0	BD	555406
PD-1	PE	J105	2.0	Invitrogen	12-2799-42
CD95	PeCF594	DX2	1.0	BioLegend	305633
NKG2D	APC	1D11	10.0	BD	558071
CD8	Alexa 700	RPA-T8	1.0	BioLegend	301027
CD3	BV786	UCHT1	2.0	BioLegend	300472
CD56	BV605	NCAM	1.0	BioLegend	318333
CD6	BV650	M-T605	2.0	BD	743448
CD57	PerCP Cy5.5	HNK-1	2.0	BioLegend	359621
NKG2A	BV421	131411	1.0	BD	747924
CD158a	PeCy7	DX27	1.0	BioLegend	312609
CD158b	PeCy7	HP-MA4	1.0	BioLegend	339511
CD158e	PeCy7	DX9	1.0	BioLegend	312720

Beckton Dickinson and Company, NJ, USA; Biolegend, CA, USA; Invitrogen, MA, USA;
Miltenyi Biotec, North Rhine-Westphalia, Germany

Table S8: Flow cytometry antibodies for T cell subsets

Antibody	Fluorochrome	Clone	Amount (uL)	Vendor	Catalog #
CD25	FITC	m-A251	1.0	BioLegend	356105
PD-1	PE	J105	2.0	Invitrogen	12-2799-42
CD95	PeCF594	DX2	1.0	BioLegend	305633
TCR a/b	PeCy 7	IP26	1.0	BioLegend	306719
CD127	APC	eBioRDR5	5.0	Invitrogen	17-1278-42
CD8	Alexa 700	RPA-T8	1.0	BioLegend	301027
TCR g/d	APC-Vio 770	11F2	2.0	Miltenyi	130-113-501
TIM 3	BV421	F38-2E2	5.0	BioLegend	345007
CD4	BV510	RPA-T4	2.0	BioLegend	300545
CD45RA	BV605	HL100	2.0	BioLegend	304133
CD6	BV650	M-T605	2.0	BD	743448
CCR7	BV711	G043-H7	5.0	BioLegend	353227
CD3	BV786	UCHT1	2.0	BioLegend	300472

Beckton Dickinson and Company, NJ, USA; Biolegend, CA, USA; Invitrogen, MA, USA; Miltenyi Biotec, North Rhine-Westphalia , Germany

Table S9: Mass cytometry NK phenotype panel

Tag	Antibody	Clone	Manufacturer	Cat.no
89Y	CD45	HI30	Fluidigm	3089003B
116Cd	HLA-DR	L243	Biolegend	307651
141 Pr	CD3	UCHT1	Fluidigm	3141019B
142Nd	CD19	HIB19	Fluidigm	3142001B
143Nd	CD127/ IL-7Ra	A019D5	Fluidigm	3143012B
144Nd	CD69	FN50	Fluidigm	3144018B
145Nd	CD4	RPA-T4	Fluidigm	3145001B
146Nd	CD8	RPA-T8	Fluidigm	3146001B
147Sm	CD336/NKp44	253415	Biolegend	325121
148Nd	CD278/ ICOS	C398.4A	Fluidigm	3148019B
149Sm	CD25	2A3	Fluidigm	3149010B
150Nd	FceRI	AER-37(CRA-1)	Fluidigm	3150027B
151Eu	CD2	TS1/8	Fluidigm	3151003B
152Sm	Granzyme B	GB11	Novus Bio	NBP1-50071
153Eu	CD62L	DREG-56	Fluidigm	3153004B
154Sm	TIGIT	MBSA43	Fluidigm	3154016B
155Gd	CD279/PD-1	EH12.2H7	Fluidigm	3155009B
156Gd	CD85j	GIH/75	Fluidigm	3156020B
158Gd	CD27	L128	Fluidigm	3158010B
159Tb	CD337/NKp30	Z25	Fluidigm	3159017B
160Gd	CD14	M5E2	Fluidigm	3160001B
161Dy	CD158a,h/ KIR2DL1/DS1	MM0438-11G	Novus Bio	NBP2-11758
162Dy	CD335/NKp46	BAB281	Fluidigm	3162021B
163Dy	CD56	NCAM16.2	Fluidigm	3163007B
164Dy	CD161	HP-3G10	Fluidigm	3164009B
165Ho	TIM-3	B27	Biolegend	345019
166Er	CD314/NKG2D	ON72	Fluidigm	3166016B
167Er	NKB1	DX9	Fluidigm	3167013B
168Er	Ki-67	SK1	Fluidigm	3168007B
169Tm	CD159a/NKG2A	ON72	Fluidigm	3169013B
170Er	CD152/CTLA-4	14D3	Fluidigm	3170005B
171Yb	CD226	DX11	Fluidigm	3171013B
172Yb	NKG2C	134522	R&D	MAB1381
173YB	CD158b	DX27	Fluidigm	3173010B
174Yb	TRAIL	R4-01	Novus Bio	NBP1-45027
175Lu	Perforin	B-D48	Fluidigm	3175004B
176Yb	CD57	HCD57	Fluidigm	3176019B
209Bi	CD16	3G8	Fluidigm	3209002B

Biolegend, CA, USA; Fluidigm, CA, USA; Novus Biologicals, CO, USA; R&D systems, MN, USA
